# Adolescent nutrition in Nigeria: a systematic review

**DOI:** 10.1017/jns.2024.34

**Published:** 2024-09-18

**Authors:** Theophilus Sunday Gabriel, Mohammed Kasim, Francis Adah Oluma, Taulant Muka, Erand Llanaj

**Affiliations:** 1 Faculty of Health Sciences, University of Debrecen, Nyíregyháza, Hungary; 2 Department of Animal Health and Production Technology, Yobe State College of Agriculture, Science and Technology Gujba, Damaturu, Yobe, Nigeria; 3 Epistudia, Bern, Switzerland; 4 Department of Molecular Epidemiology, German Institute of Human Nutrition Potsdam-Rehbrücke, Nuthetal, Germany; 5 German Centre for Diabetes Research (DZD), München, Germany

**Keywords:** Adolescents, Anthropometry, Nigeria, Nutrition, Systematic review

## Abstract

In this systematic review, we scrutinise adolescent nutrition in Nigeria, focusing on dietary patterns, intake, and nutritional status. Through a systematic examination of observational studies across three major databases, we analysed data from 67,844 adolescents. Our exploration revealed 102 studies, predominantly cross-sectional, addressing various nutritional dimensions. However, only 13% of these studies demonstrated low risk of bias, with none offering national representation and most concentrated in specific, school-based regions. The findings underscore a complex nutritional landscape with widespread malnutrition and highlight the critical need for high-quality, comprehensive data. The dominance of cross-sectional designs and regional biases in existing research calls for cautious interpretation and suggests a pressing need for more robust, nationally representative studies to guide future nutritional interventions and policy-making in Nigeria.

## Introduction

Promoting health and well-being of adolescents in low- and middle-income countries (LMICs) is a global health priority, given that they account for more than 90% of the world’s 1.2 billion adolescent population.^(1)^ This age group, spanning from 10 to 19 years, represents a unique life period marked by physical, cognitive, psychosocial and emotional changes. The health needs of adolescents, particularly those living in LMIC, are distinct from other age groups and overlooking this can have lasting impacts on growth and development of future generations. Thus, adolescence offers a window of opportunity for interventions that can greatly shape health outcomes later in life and foster a sustainable and equitable future for societies.

Recognising the importance of this life phase, the United Nations Sustainable Development Goals (SDGs) explicitly mention adolescence in 12 health-related SDG indicators.^(2)^ Research has further reinforced the role of nutrition on adolescent growth and development, the influence of the food environment on their food choices and which interventions might lead to healthier nutrition and growth.^(3)^ These efforts have renewed the interest in investing in nutrition - a major modifiable risk factor with significant implications for public health.^(4)^ Indeed, large prospective human studies have shown that nutritional status in adolescence is a strong predictor of adult cardiovascular health.^(5)^

However, in LMIC settings usually, adolescents have limited control over their food choices. In Nigeria, the limited investment in nutritional research, particularly regarding adolescent nutrition, poses a great challenge in monitoring the state and trajectories of nutrition indicators across regions and over time.^(6)^ Nevertheless, such nutrition surveillance data can be a valuable guide for formulating policies and interventions tailored to this demographic group. According to the latest Global Nutrition Report 2022, Nigeria has only made slight progress towards diet-related non-communicable disease (NCD) targets.^(7)^ Given the effects of the post-pandemic era and the ongoing influence of food prices on household finances, along with the challenges pertaining to food affordability, accessibility of nutritious diets and livelihoods, a careful assessment of the state of adolescent nutrition in Nigeria is warranted.^(8)^

Therefore, high-quality evidence on Nigerian adolescent nutrition can inform tailored solutions and support better nutrition and health. Findings and insights can also be used as an invaluable tool in shaping the development and establishment of evidence-based guidelines and nutrition surveillance systems for monitoring dietary risks among Nigerian adolescents. Taking this into account, we conducted a systematic review aiming to comprehensively summarise and examine the state of adolescent nutrition in Nigeria, including dietary intake profile, habits and nutritional status.

## Methods

This work was carried out in accordance with established guides for conducting evidence syntheses for medical and health research,^(9,10)^ as well as PRISMA guidelines for reporting findings from systematic reviews and meta-analyses.^(11)^ We systematically searched three electronic databases namely: PubMed, Web of Science and Google Scholar, from inception to March 6, 2023. The protocol of this work is registered in the international prospective register of systematic reviews (PROSPERO) with identification code **CRD42023399668**.

### Selection of studies

Studies were considered for inclusion if they met the following criteria: (i) carried out in Nigeria, (ii) included adolescents between the ages of 10–19 years and (iii) reported dietary intake, patterns, adherence to a healthy diet, and/or nutritional status (i.e. anthropometry and other related measures). Only observational studies were considered for inclusion. There have been recent proposals^(12)^ to extend the definition of adolescence from 10–19 years to 10–24 years, noting delays in the transition age to adult roles (e.g. marriage and parenthood) in many societies as the main motivation. However, in this work, we use the definition of 10–19 years. Studies were not included if: (i) they were not conducted in Nigeria and (ii) did not report any nutrition-related indicator (e.g. nutritional status, dietary intake or adherence to healthy diet standards). In addition, we did not consider case studies/reports, letters to the editor, conference proceedings, posters, abstracts, reviews or preprints.

### Data extraction

Three reviewers independently evaluated the titles and abstracts according to the inclusion and exclusion criteria. For each eligible study, three reviewers assessed the full-text. In cases of disagreement, a decision was made by consensus or, when necessary, a fourth reviewer was consulted. Information was extracted from studies in triplicate and was categorised according to the following variables: study design, study area (region, state), target population, age, sex, sample size, setting, dietary assessment tool used, journal details, intake of macro- and micro-nutrients, etc.

### Quality assessment of included studies

We evaluated the methodological rigour of included studies using the Joanna Briggs Institute (JBI) corresponding tool for assessing methodological quality of studies and provided answers to the relevant questions, based on study design.^(13)^ Detailed assessment can be found in supplementary Table S4. The methodological quality was rated on a scale of maximum 8 points. Based on this evaluation, studies were classified as ‘*low risk of bias*’ (>= 7 points), ‘*some concerns*’ for bias (>=4 points), and ‘*high risk of bias*’ (< 4 points). We included all studies in the synthesis, irrespective of their JBI evaluation classification.

### Data synthesis

We synthesised the extracted data from each study and described the information on dietary assessment tool and/or nutritional status (e.g. anthropometry classification) for the target group (10–19 years) with no discrimination to setting (e.g. in school or out of school), location or sex. We extracted primary and composite anthropometric parameters, and dietary intake in comparison to internationally established reference values and the primary aims of each included study were reported. Nutrient, energy and other metrics were converted to same units (e.g. energy in kcal, protein intake in grams, etc.). We also report the food and food group consumption and dietary habits among Nigerian adolescents. In order to show the nutritional status trends over time a summary of results from studies reporting anthropometric indicators ordered by year of publication was produced using R Studio 2022.07.2 Build 576 (Fig. 2). Data used to produce this figure can be found in supplementary Table S3.

**Fig. 1.**
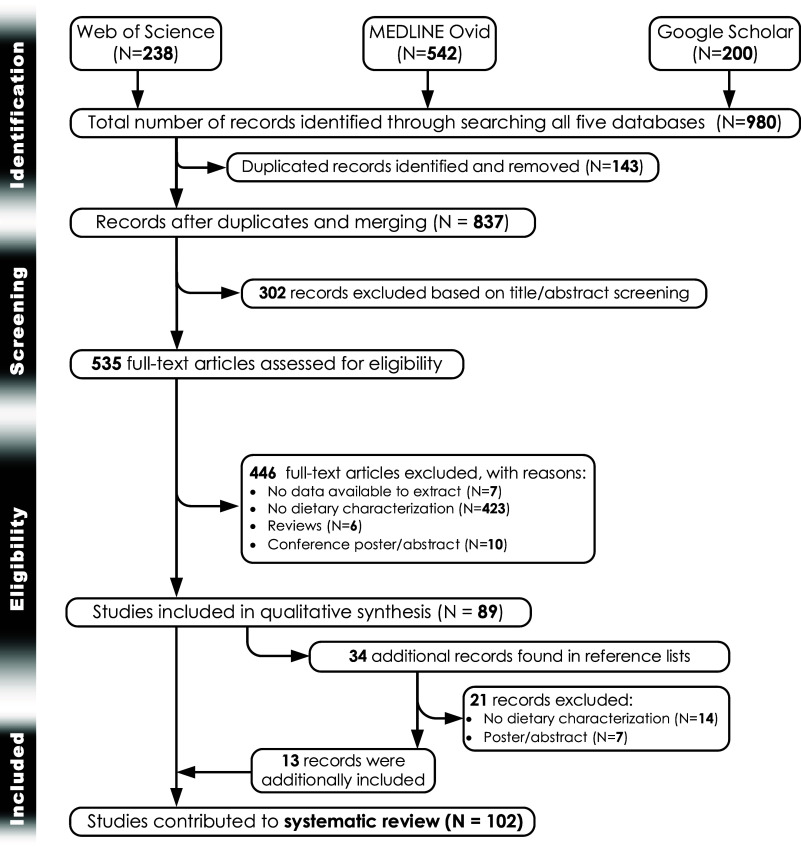
PRISMA flowchart.^(14)^

## Results

Based on the systematic search (Fig. 1), we retrieved 238 articles from Web of Science and 542 from PubMed search engines. In Google Scholar, the first 200 results were considered. Supplementary Table S1 outlines the search strategy and strings used for each database. Based on the bibliographic searches, 980 records were retrieved in total. Following deduplication and merging 837 studies were left. 302 studies were excluded after title and abstract screening, leaving 535 records for full-text screening. Of those, 446 studies were excluded for various reasons. Eventually, 89 studies were included for qualitative synthesis. In addition, 34 studies were found after screening the reference lists of the 89 studies included in the qualitative synthesis. Of these 34 records, only 13 studies were eligible to be added to the previous 89 studies, bringing the number of studies contributing to the systematic review to 102 records.

**Fig. 2.**
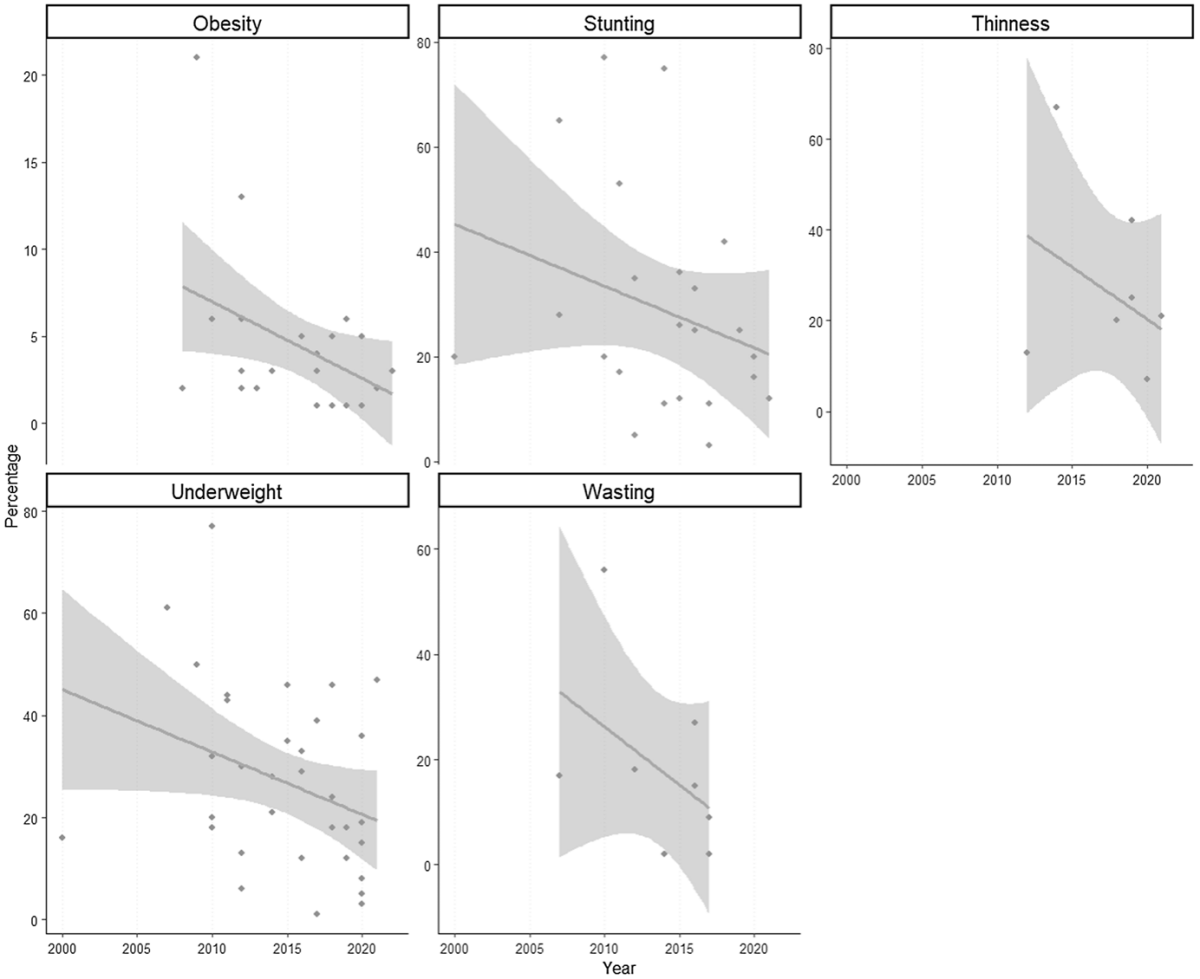
Summary of the individual studies reporting anthropometric indicators over time.

Table 1 shows the characteristics of included studies. A total of 102 cross-sectional studies have been examined and reported. Sample sizes ranged from as small as 22 to as large as 7,625. Approximately half of the study sites, representing (n = 51), were located in the south-western region of the country. Conversely, the north western region was the least represented with merely 3.9% of the studies conducted there (4 out of 102 studies). A visual map with study locations and population density of all included studies is available in supplementary Fig. S1. The majority of studies (86%) included both male and female participants. Educational institutions were predominantly utilised as the research setting in over 70% (77 out of 102) of the studies. Substantial heterogeneity was observed with regards to the methodological approach to dietary assessment and anthropometric classification tools adopted in these studies. Questionnaires were the most widely employed data collection instrument, featured in nearly 81 out of 102 studies. Based on the JBI tool evaluation, 13 studies were classified as ‘low risk of bias’, representing 12.7% of the total 102 studies. Conversely, 46 studies, accounting for 45.1%, were assessed as having ‘some concerns’ for bias, while 43 studies, or 42.2%, were rated as at ‘high risk of bias’. The most commonly used dietary assessment tool was a questionnaire. Other tools included the 24-hour dietary recall, food frequency questionnaire, and diet diversity score. The administration method was predominantly self-administered or researcher-administered. Validation: There was a mixed validation status for the instruments used. While many of the questionnaires were not validated, a minority of studies used validated tools. Most of studies (84.3% or 86/102) were published in journals that were not indexed and consequently had no impact factor at the time of publication.

**Table 1.** Study characteristics of the studies included in the systematic review

Ref.	Author, year	Location	Target population	Age, mean (SD)	Sex	Setting	Sample size	Dietary assessment instrument (validation/ administration)	Intake quantified?	Risk of bias	Journal indexed in MJL
^(15)^	Abdulkarim *et al.*, 2014	Abuja	Adolescents only	14.4 ± 1.9	♀♂	Primary school	1700	Questionnaire (Q), Not validated (NV), self-administered (SA)	No	Some concerns	No
^(16)^	Abidoye and Akande, 2000	Lagos State.	Adolescent/Children	NR	♀♂	Primary school	120	Q (NV/SA)	No	High Risk	No
^(17)^	Adebimpe, 2019	Osogbo	Adolescents only	14.6 ± 1.7	♀♂	Community	480	24 HDR (NV/SA)	Yes	High Risk	Yes
^(18)^	Adeomi *et al.*, 2019	Ile-Ife, Osun state.	Adolescents only	14.4 ± 2.0	♀♂	Community	313	Q (NV/SA)	No	Low Risk	No
^(19)^	Adeomi *et al.*, 2022a	Southwest, Nigeria	Adolescents only	♀14.4 ± 1.9 & ♂14.8 ± 2.1	♀♂	Community	400	Q (Validated (V)/SA)	Yes	Low Risk	No
^(20)^	Adeomi *et al.*, 2022b	Osun and Gombe states	Adolescent/Children	NR	♀♂	Rural and urban	1200	Food frequency questionnaire (FFQ) (V/SA)	Yes	Low Risk	Yes
^(21)^	Adeomi *et al.*, 2022c	Osun and Gombe states.	Adolescent/Children	NR	♀♂	Rural and urban	1200	Q(NV/Researcher-administered (RA))	Yes	Some concerns	Yes
^(22)^	Adesina *et al.*, 2012	Port- Harcourt	Adolescents only	14.5 ± 2.3	♀♂	Secondary school	960	Q(NV/SA)	No	Some concerns	Yes
^(23)^	Adinma *et al.*, 2020	Nnewi, Anambra state.	Adolescents only	14.5 ± 3.0	♂	Secondary school	311	FFQ (V/RA)	Yes	High Risk	No
^(24)^	Adu *et al.*, 2009	Lagos State	Adolescent/Adults	NR	♀♂	University	100	Q (NV/SA)	Yes	Some concerns	No
^(25)^	Afolabi *et al.*, 2013	Abeokuta	Adolescents only	NR	♀♂	University	140	24-hour dietary recall (24 HDR) (NV/SA)	Yes	High Risk	No
^(26)^	Agofure *et al.*, 2021	Delta State	Adolescents only	NR	♂	Secondary school	201	Q(NV/SA)	No	Some concerns	No
^(27)^	Agoreyo *et al.*, 2002	Benin City, Edo state	Adolescents only	NR	♂	University	500	Q(NV/SA)	Yes	High Risk	No
^(28)^	Ajuzie *et al.*, 2018	Ogun State.	Adolescent/Children	NR	♀♂	Primary school	1200	Q(NV/RA)	Yes	High Risk	No
^(29)^	Akinbodewa *et al.*, 2020	Ondo state	Adolescents only	13.0 ± 2.0	♀♂	Primary school	160	Q(NV/SA)	No	Low Risk	No
^(30)^	Akinlade *et al.*, 2014	Oyo state	Adolescents only		♀♂	Secondary school	821	Q(V/SA)	Yes	Some concerns	No
^(31)^	Akinola *et al.*, 2022	Lagos state	Adolescents only	14.3 ± 2.1	♀♂	Secondary school	1120	FFQ (NV/SA)	Yes	Low Risk	No
^(32)^	Akinyemi *et al.*, 2009	Lagos state.	Adolescents only	NR	♂	Secondary school	40	Q (NV/SA)	Yes	High Risk	No
^(33)^	Ansa *et al.*, 2008	Calabar Cross river state.	Adolescents only	NR	♀♂	Secondary school	1000	Q (NV/SA)	No	High Risk	No
^(34)^	Anyika *et al.*, 2009	Abia State	Adolescents only	NR	♂	Secondary school and university	160	Q (NV/SA)	Yes	High Risk	No
^(35)^	Atawodi *et al.*, 2015	Kaduna state.	Adolescent/Children	NR	♀♂	Secondary school	141	Q (NV/SA)	No	High Risk	No
^(36)^	Ayogu *et al.*, 2016	Nsukka, Enugu state.	Adolescents only	NR	♀♂	Secondary school	400	Q(NV/SA)	Yes	Low Risk	No
^(37)^	Ayogu *et al.*, 2018	Enugu state	Adolescent/Children	NR	♀♂	Primary and secondary school	450	Q (V/RA)	Yes	Low Risk	No
^(38)^	Ayogu *et al.*, 2019	Ede-Oballa, Enugu state	Adolescent/Children	NR	♀♂	Primary and secondary school	450	Diet Diversity Score (DDS) (NV/SA)	Yes	Some concerns	Yes
^(39)^	Ayogu *et al.*, 2021	South-east Nigeria.	Adolescents only	NR	♀♂	Rural	401	Q (NV/SA)	No	Low Risk	Yes
^(40)^	Bamidele *et al.*, 2016	Lagos State	Adolescent/Children	NR	♀♂	Primary school	529	Q (NV/RA)	Yes	Some concerns	No
^(41)^	Charles *et al.*, 2020	Borno State	Adolescents only	NR	♂	Secondary school	612	Q (NV/RA)	No	Some concerns	No
^(42)^	Cole *et al.*,1997	Ibadan	Adolescents only	NR	♀♂	Secondary school	22	Q (NV/SA)	Yes	High Risk	No
^(43)^	Darling *et al.*, 2020	Sub-Saharan Africa including Nigeria (Ibadan)	Adolescents only	NR	♀♂	Secondary school	7625	Q (NV/RA)	Yes	Low Risk	No
^(44)^	Ekekezie *et al.*, 2012	Lagos State	Adolescent/Children	NR	♀♂	Primary school	529	Q (NV/RA)	No	Some concerns	No
^(45)^	Elizabeth *et al.*, 2009	Makurdi, Benue State	Adolescent/Children	NR	♀♂	Secondary school	600	Q (NV/SA)	No	Some concerns	No
^(46)^	Ene-Obong *et al.*, 2003	Enugu State	Adolescents only	NR	♀♂	Community	135	Q (NV/SA)	Yes	Some concerns	Yes
^(47)^	Ene-Obong *et al.*, 2012	Lagos, Rivers, Enugu and Abia state.	Adolescent/Children	NR	♀♂	Community	1,599	Q (V/RA)	No	High Risk	Yes
^(48)^	Eneobong, 1993	Nsukka, Enugu	Adolescents only	NR	♀♂	Community	50	Q (NV/SA)	Yes	High Risk	Yes
^(49)^	Erinoso *et al.*, 1992	Olodo, Oyo state	Adolescent/Children	NR	♀♂	Community	400	Q (NV/SA)	Yes	High Risk	Yes
^(50)^	Esimai *et al.*, 2015	Port Harcourt	Adolescents only	♂:12.5 ± 0.1; ♀:12.6 ± 0.1	♀♂	Secondary school	182	Q (NV/SA)	No	Low Risk	No
^(51)^	Essien *et al.*, 2014	Sokoto	Adolescent/Adults	♂:18.0 ± 1.9; ♀:15.7 ± 1.2	♀♂	Secondary school	240	Q (V/SA)	No	High Risk	No
^(52)^	Eze *et al.*, 2017	Enugu State	Adolescent/Adults	NR	♀♂	Secondary school	2616	Q (NV/SA)	No	Some concerns	Yes
^(53)^	Fadipe *et al.*, 2017	Lagos, state.	Adolescent/Adults	21.4	♀♂	University	1,054	Q (NV/SA)	Yes	Some concerns	Yes
^(54)^	Fagbamigbe *et al.*, 2019	Oyo state	Adolescent/Children	NR	♀♂	Secondary school	390	Q (NV/RA)	No	Some concerns	Yes
^(55)^	Folashade *et al.*, 2016	Ogun State	Adolescents only	NR	♀♂	Secondary school	572	Q (NV/SA)	No	Low Risk	No
^(56)^	Funke and Ajayi, 2007	Osun State	Adolescents only	NR	♀♂	Secondary school	450	Q (NV/SA)	Yes	Some concerns	No
^(57)^	Goon *et al.*, 2011	Makurdi, Benue State	Adolescents only	NR	♀♂	Secondary school	2015	Q (NV/SA)	No	Some concerns	Yes
^(58)^	Henry-Unaeze *et al.*, 2011	Nnewi, Abia state	Adolescents only	NR	♀♂	Secondary school	200	Q (NV/SA)	No	High Risk	No
^(59)^	Ikorok *et al.*, 2012	Akwa Ibom	Adolescents only	NR	♀♂	Secondary school	450	Q (V/RA)	No	Some concerns	No
^(60)^	Ikujenlola and Adekoya, 2020	Osun state	Adolescents only	NR	♂	University	200	FFQ (V/SA)	Yes	High Risk	Yes
^(61)^	Iyalomhe *et al.*, 2018	Ekpoma, Edo state	Adolescents only	NR	♀♂	Secondary school	400	Q (V/SA)	Yes	Some concerns	No
^(62)^	Kayode *et al.*, 2020	Ede, Osun State	Adolescent/Adults	19.8 ± 1.3	♀♂	University	268	FFQ (NV/SA)	Yes	Some concerns	No
^(63)^	Kelvin and Sanusi, 2016	EKITI state	Adolescents only	14.4 ± 1.9	♀♂	Secondary school	789	24 HDR (NV/RA)	Yes	Some concerns	No
^(64)^	Kola-Raji *et al.*, 2017	Ibadan, Oyo state.	Adolescents only	♂:12.9 ± 2.05; ♀:14.0 ± 1.49	♀♂	Secondary school	490	Q (NV/RA)	No	High Risk	No
^(65)^	Lateef *et al.*, 2016	Kwara state	Adolescents only		♀♂	Secondary school	515	FFQ (NV/SA)	Yes	High Risk	No
^(66)^	Nnanyelugo *et al.*, 1982	Anambra	Adolescent/Children	NR	♀♂	Community	2036	Q (NV/SA)	Yes	Some concerns	No
^(67)^	Nwokoro *et al.*, 2006	Benin city, Edo state.	Adolescent/Adults	NR	♀♂	Secondary school	2012	Q (NV/SA)	No	High Risk	No
^(68)^	Ogechi *et al.*, 2007	Umuahia, Abia State	Adolescents only	NR	♀♂	Secondary school	190	Q (NV/SA)	Yes	High Risk	No
^(69)^	Ogechi *et al.*, 2012	Umuahia, Abia State	Adolescents only	NR	♀♂	Secondary school	416	Q (NV/SA)	Yes	High Risk	No
^(70)^	Ogunkunle and Oludele, 2013	Ila Orangun, Osun state	Adolescents only	NR	♀♂	Secondary school	302	FFQ (NV/RA)	Yes	High Risk	No
^(71)^	Ogunsile,2012	Ekiti state	Adolescents only	NR	♀♂	Secondary school	128	Q (NV/SA)	Yes	Low Risk	No
^(72)^	Oguntona and Kanye, 1995	Ogun State	Adolescents only	NR	♀♂	Secondary school	187	Q (NV/SA)	Yes	High Risk	No
^(73)^	Okeke *et al.*, 1989	Anambra	Adolescent/Children	NR	♀♂	Community	387	Q (NV/SA)	Yes	High Risk	Yes
^(74)^	Okoro *et al.*, 2016	Ibadan, Oyo state	Adolescent/Children	13.6 ± 3.6	♀♂	Secondary school	464	FFQ (V/SA)	Yes	Some concerns	Yes
^(75)^	Okpokowuruk *et al.,* 2017	Uyo, Akwa Ibom	Adolescent/Children	NR	♀♂	(I+II)	195	Q (NV/SA)	No	Some concerns	No
^(76)^	Olatona *et al.*, 2018	Lagos state.	Adolescent/Adults	20.3 ± 3.5;	♀♂	University	506	FFQ (V/RA)	Yes	High Risk	Yes
^(77)^	Olatona *et al.*, 2020	Lagos state	Adolescents only	3.6 ± 2.3	♀♂	Community	682	Q (NV/RA)	Yes	High Risk	No
^(78)^	Olatona *et al.*, 2022	Lagos State.	Adolescents only	13.8 ± 1.7	♀♂	Secondary school	397	Q (NV/SA)	Yes	High Risk	Yes
^(79)^	Olorunfemi *et al.*, 2019	Kaduna, State	Adolescents only	14.3 ± 2.8	♀♂	University	50	Q (V/RA)	No	Some concerns	No
^(80)^	Olumakaiye *et al.*, 2010	Osun State	Adolescents only	NR	♀♂	Secondary school	401	Q (V/SA)	Yes	High Risk	Yes
^(81)^	Olumakaiye, 2013	Osun State	Adolescent/Children	NR	♀♂	Secondary school	600	24 HDR (V/SA)	Yes	High Risk	No
^(82)^	Olumuyiwa *et al.*, 2012	Ile-Ife, Osun State	Adolescent/Children	NR	♀♂	Primary school	160	Q (NV/SA)	Yes	High Risk	No
^(83)^	Oluyinka *et al.*, 2020	Iwo Osun State	Adolescents only	NR	♀♂	University	216	Q (NV/SA)	Yes	Some concerns	No
^(84)^	Omigbodun *et al.*, 2010	Ibadan, Oyo state	Adolescents only	NR	♀♂	Secondary school	1799	Q (NV/SA)	Yes	Some concerns	Yes
^(85)^	Omobuwa *et al.*, 2014	Ibadan, Oyo state	Adolescents only	15.67 ± 1.25	♀♂	Secondary school	93	Q (NV/SA)	Yes	Some concerns	No
^(86)^	Omuemu and Oko-Oboh, 2015	Benin city, Edo state	Adolescents only	15.4 ± 3.6	♀♂	Secondary school	797	Q (NV/RA)	Yes	High Risk	No
^(87)^	Omuemu *et al.*, 2010	Benin-city	Adolescents only	15.3 ± 1.9	♀♂	Community	300	Q (NV/RA)	No	High Risk	Yes
^(88)^	Onabanjo and Balogun, 2014	Ogun State	Adolescents only	16.0 ± 2.3	♀♂	Secondary school	127	Q (V/RA)	Yes	Low Risk	No
^(89)^	Onimawo *et al.*, 2010	Abia state	Adolescent/Children	NR	♀♂	Primary school	249	FFQ (NV/SA)	Yes	Some concerns	No
^(90)^	Oninla *et al.*, 2007	Ile-Ife,Osun State	Adolescent/Children	10.2 ± 2.7	♀♂	Rural	749	Q (NV/SA)	No	Some concerns	Yes
^(91)^	Onofiok *et al.*, 1996	Emene, Nsukka	Adults/Adolescents/Children	NR	♀♂	Community	1030	Q (NV/SA)	Yes	High Risk	No
^(92)^	Onuoha and Eme, 2013	Aba, Abia state	Adolescents only	4.6 ± 1.8	♀♂	Secondary school	600	Q (NV/SA)	Yes	High Risk	No
^(93)^	Onyechi and Okolo, 2009	Enugu, State	Adolescents only	NR	♀♂	University	620	Q (V/SA)	Yes	High Risk	No
^(94)^	Onyiriuka *et al.*, 2013	Benin City, Edo state	Adolescents only	14.8 ± 1.9	♂	Community	2,097	Q (NV/SA)	Yes	Some concerns	No
^(95)^	Onyiriuka *et al.*, 2013	Edo State	Adolescents only	14.5 ± 2.0	♂	Secondary school	2,304	Q (NV/SA)	Yes	Some concerns	No
^(96)^	Onyiriuka *et al.*, 2013	Edo State	Adolescents only	NR	♂	Secondary school	2 097	Q (NV/SA)	Yes	Some concerns	No
^(97)^	Opara and IEE, 2010	UYO, Akwa Ibom state	Adolescent/Children	NR	♀♂	Community	500	Q (NV/SA)	No	Some concerns	No
^(98)^	Oranusi *et al.,* 2007	Zaria, Kaduna state	Adolescent/Adults	NR	♀♂	Community	44	Q (NV/SA)	Yes	High Risk	No
^(99)^	Orisa and Wordu, 2021	Portharcourt, Rivers state	Adolescents only	NR	♂	Secondary school	236	Q (V/RA)	Yes	High Risk	No
^(100)^	Otekunrin and Otekunrin,2022	Ogun and Oyo States	Adolescents only	13.6 ± 2.7	♀♂	Community	160	Q (NV/SA)	Yes	Some concerns	No
^(101)^	Otemuyiwa and Adewusi, 2012	Osun and Ondo State	Adolescent/Adults	NR	♀♂	University	402	FFQ (NV/SA)	Yes	High Risk	Yes
^(102)^	Otuneye *et al.*, 2017	FCT – Abuja	Adolescents only	14.4 ± 1.9	♀♂	Secondary school	1550	Q (NV/SA)	Yes	Some concerns	No
^(103)^	Samuel *et al.,* 2015	Ibadan, Oyo state	Adolescents only	NR	♀♂	Community	190	FFQ (NV/RA)	Yes	High Risk	No
^(104)^	Samuel *et al.,* 2021	Ibadan, Oyo state	Adolescents only	16.9 ± 1.9	♀♂	Community	300	FFQ (NV/SA)	Yes	Some concerns	No
^(105)^	Sanusi *et al.*, 2021	Ibadan, Oyo state	Adolescents only	NR	♀♂	Community	433	24 HDR (NV/RA)	Yes	High Risk	Yes
^(106)^	Senbanjo *et al.*, 2011	Abeokuta, Ogun state	Adolescent/Children	12.2 ± 3.4	♀♂	Secondary school	570	Q (NV/SA)	No	Some concerns	Yes
^(107)^	Senbanjo *et al.*, 2014	Abeokuta, Ogun state	Adolescent/Children	NR	♀♂	Secondary school	570	Q (NV/SA)	No	Some concerns	Yes
^(108)^	Shapu *et al.*, 2020	Borno State	Adolescents only	NR	♂	Secondary school	612	Q (NV/SA)	No	Some concerns	Yes
^(109)^	Shokunbi and Ukangwa, 2021	Ogun State	Adolescents only	NR	♀♂	Secondary school	488	Q (NV/SA)	Yes	Some concerns	Yes
^(110)^	Sholeye *et al.*, 2018	Sagamu, Ogun state	Adolescents only	15.6 ± 1.2	♀♂	Secondary school	620	Q (NV/SA)	No	High Risk	No
^(111)^	Silva *et al.*, 2017	Lagos State	Adolescents only	12.4	♀♂	Secondary school	220	Q (NV/RA)	Yes	Some concerns	No
^(112)^	Tassy *et al.*, 2021	Ibadan, Osun state	Adolescent/Children		♀♂	Community	955	24 HDR (NV/RA)	Yes	Some concerns	Yes
^(113)^	Uba *et al.*, 2020	Bauchi State	Adolescents only	15.9 ± 0.9	♂	Secondary school	250	Q (V/SA)	No	Some concerns	No
^(114)^	Umeokonkwo *et al.*, 2020	Ebonyi State	Adolescent/Children	NR	♀♂	Primary school	780	Q (NV/RA)	No	Some concerns	Yes
^(115)^	Wariri *et al.,* 2020	Gombe State and Uyo, Akwa Ibom state	Adolescents only	NR	♀♂	Community	2100	Q (V/RA)	No	Some concerns	Yes
^(116)^	Yunusa *et al.*, 2014	Nigeria	Adolescents only	NR	♀♂	Community	270	Q (NV/SA)	Yes	High Risk	No

*Note*: ♀, Male; ♂, Female; NR, Not Reported.

Table 2 provides a summary of anthropometric findings of nutritional epidemiology studies conducted in Nigeria among adolescents. Out of the total, sixty-six studies documented either primary or composite anthropometric measures or a combination thereof. Composite anthropometric measures encompassed various indicators such as underweight, overweight, stunting, obesity, thinness and wasting. Of note, 77.2% of the studies reported different manifestations of undernutrition, such as stunting, wasting and/or underweight. Furthermore, 68.2% of the studies indicated the prevalence of overweight and obesity, which constitute forms of over nutrition. Additionally, the height-for-age z-score (HAZ) and weight-for-age *z*-score (WAZ) were reported in about 39% of the studies (25 out of 66). The overall range for body mass Index (BMI, in kg/m^2^) across all studies that reported it, ranged from 15.7 to 24.6. Height (in metres) varied within a range of 1.2–1.7 and weight (in kilograms) ranged from 19 to 70. With regard to the prevalence of simple nutritional status phenotypes (expressed as percentages), the overall range was as follows: underweight from 2.5% to 78.3%; overweight from 1.4% to 25.2%; stunting from 2.5% to 77.1%; wasting from 1.7% to 56%; and obesity from 0.2% to 21%.

**Table 2.** Anthropometric findings from Nigerian nutritional epidemiology studies

Ref.	Author, year	Primary anthropometric measures	Composite anthropometric measures (%)
^(26)^	Agofure *et al.*, 2021	NR	Underweight: 46.8
			Normal weight: 31.8
			Overweight: 21.4
^(104)^	Samuel *et al.*, 2021	NR	Stunting (males): 20.5
			Stunting (females): 6.8
			Stunting: 12.1
			Overweight/obese: 9.5
^(74)^	Okoro *et al.*, 2016	Average BMI (kg/m^2^): 17.8	Underweight occurrence was high
^(60)^	Ikujenlola and Adekoya, 2020	BMI(kg/m^2^) : Private University 22.2 ± 3.5, Public University 21.9 ± 4.2	Normal weight: 67.5
			Underweight: 15
			Overweight: 12
			Obesity: 4.5
^(18)^	Adeomi *et al.*, 2019	NR	Overweight and obesity: 10.2
			Underweight: 12.1
			Overweight: 8.9
			Obesity: 1.3
			Overweight/Obesity (females): 12.5
			Overweight and Obesity (males): 7
^(102)^	Otuneye *et al.*, 2017	NR	Overweight: 12.6
			Stunting: 11.2
			Obesity: 2.8
			Wasting: 1.7
^(21)^	Adeomi *et al.*, 2022c	NR	Thinness: 10.3
			Overweight/Obesity: 11.4
			Rural; Thinness: 12.8
			Urban; Thinness: 7.7
			Rural; Obesity/Overweight: 7
			Urban; Obesity/Overweight: 15.8
^(20)^	Adeomi *et al.*, 2022b	NR	Thinness: 10.3
			Overweight/Obesity: 11.4
^(89)^	Onimawo *et al.*, 2010	Triceps (m) (mean ± standard deviation): 0.0043 ± 1.8; Height (m): 1.3 ± 9.7; Weight (Kg): 28.4 ± 5.8; Subscapular (m):0.0043 ± 1.4; Suprailiac (m): 0.0035 ± 1.4; waist circumference (m): 0.0045 ± 1.8	Stunting: 77.1
			Wasting: 56
			Underweight: 77.1
^(35)^	Atawodi *et al.*, 2015	NR	Underweight: 35
			Stunting: 26.4
^(36)^	Ayogu *et al.*, 2016	12-14yrs.: Weight (Kg): 48.2; Height(m): 1.5	Stunting: 33.3; Thinness: 31
		15–18 yrs.: Weight (Kg): 56.3; Height (m): 1.6	
^(32)^	Akinyemi *et al.*, 2009	Weight (Kg): 58.9	NR
		Height(m): 1.7	
		BMI(Kg/m^2^): 21.4	
^(45)^	Elizabeth *et al.*, 2009	BMI(Kg/m^2^): 16.3 ± 2.4 for males and for females, 16.7 ± 2.7	L.G.E.A Wurukum: Underweight: 73.3
			C.A.C Wadata: Underweight: 78.3
			B.S.Urban: 34.16 Underweight: 34.2
			Urban.A.M: Underweight: 38.3
			Nativity School: underweight: 28.3
^(17)^	Adebimpe, 2019	NR	3.8 Obese/Overweight
^(79)^	Olorunfemi *et al.*, 2019	Weight (Kg): 35.9 ± 10.4	Overweight: 6
			Obesity: 6
			Thinness: 42
			Severe thinness: 38
^(39)^	Ayogu *et al.*, 2021	NR	Male: Overweight: 3.7
			Female: Overweight: 2.8
^(47)^	Ene-Obong *et al.*, 2012	NR	Overweight: 11.4
			Obesity: 2.8
			Thinness: 13
^(53)^	Fadipe *et al.*, 2017	Weight (Kg):63.3 ( ± 11.14); Height (m) :1.7 ( ± 0.9); BMI (Kg/m^2^): 22.2 ( ± 3.6)	NR
^(54)^	Fagbamigbe *et al.*, 2019	NR	**Males:** Underweight: 19, Stunting: 27, Thinness: 27
			**Females:** Underweight: 17, Stunting: 23, Thinness: 23
^(101)^	Otemuyiwa and Adewusi, 2012	Mean BMI (kg/m^2^): Males (AAU): 23.6; Males (OAU): 22.3	Overweight: 29
			Obesity: 6
			Underweight: 13
^(76)^	Olatona *et al.*, 2018	NR	Overweight: 15
			Obesity: 5
^(93)^	Onyechi and Okolo, 2009	NR	Obesity: 21
^(24)^	Adu *et al.*, 2009	BMI(Kg/m^2^): 24.6 ± 3.3; Mid arm circumference (m): 0.3 ± 3; Weight (Kg): 64 ± 9.1; Height(m): 1.6 ± 0.23	NR
^(106)^	Senbanjo *et al.*, 2011		Stunting: 17.4
			Severely Stunted: 22.2
^(75)^	Okpokowuruk *et al.*, 2017	Mean weight (Kg): 37.4	NR
		Mean height(m): 1.4	
		Waist circumference(m): 0.6	
^(112)^	Tassy *et al.*, 2021	NR	Obesity: 1.6
			Overweight: 3.0
			Thinness: 21.0
			Stunted: 18.2
^(88)^	Onabanjo and Balogun, 2014	Weight (kg): 43.3 ± 7.8 (male 41.9 ± 8.5, female 44.6 ± 9.3); Waist (m): 0.6 ± 13.7 (male 0.6 ± 12.6, female 0.7 ± 13.9)	Underweight: 21.3; Overweight: 14.2; Waist/hip(m): 0.82 ± 0.3 (male 0.8 ± 0.3, female 0.9 ± 0.5)
^(114)^	Umeokonkwo *et al.*, 2020	NR	Underweight: 8
			Thinness: 7.2
			Stunting: 9.9
			Overweight: 1.4
			Obesity 0.7
^(40)^	Bamidele *et al.*, 2016	NR	Rural: Overweight 15.1; Underweight: 49.6; Wasting: 24.2
			Urban : Overweight: 13.2; Underweight: 15; Wasting: 13.6
^(44)^	Ekekezie *et al.,* 2012	NR	**Rural area:** Underweight: 49.6; Stunting: 50.8; Wasting: 24.2
			**Urban area :** Underweight: 15.1; Stunting: 16.6; Wasting: 13.6; Overweight: 5.1; Obesity: 13.2
^(16)^	Abidoye and Akande, 2000	NR	**Upland (urban):** Stunting: 15.8; Wasting: 3.3; Underweight: 14.2
			**Riverine (rural):** Stunting: 30; Wasting: 1.7; Underweight: 18.3
^(80)^	Olumakaiye *et al.*, 2010	NR	Underweight: 20.1 (rural 22.1 and urban 18.7)
^(107)^	Senbanjo *et al.*, 2014	NR	Stunting: 75
			Thinness: 66.7
^(83)^	Oluyinka *et al.*, 2020	NR	Underweight: 2.5
			Obesity:5
			Overweight: 25.2
^(55)^	Folashade *et al.*, 2016	NR	Wasting: 26.7
			Stunting: 24.8
^(28)^	Ajuzie *et al.*, 2018	Mean weight (Kg): 19 − 70	Underweight: 45.8
		Mean height(m): 1.2 − 1.6	
^(19)^	Adeomi *et al.*, 2022a	NR	Overweight/ Obesity: 12.8
^(23)^	Adinma *et al.*, 2020	Weight (Kg): 51.8 ± 10.4	Underweight: 7.7
		Height(m): 1.6 ± 0.1	Overweight: 9.0
		BMI (kg/m^2^): 20.1 ± 3.5	Obesity: 1.3
^(30)^	Akinlade *et al.*, 2014	Height (m): Male 1.6 ± 8.5, female 1.6 ± 6.7	BMI-for-Age percentile: 82.5: 5^th^-85^th^ percentiles (Normal); 2.2: 85^th^-95^th^ percentiles (Overweight); 2.6: 95^th^ percentile (Obesity)
		BMI (kg/m^2^): Male 19.8 ± 2.8, female 20.5 ± 3.1	
		Triceps (m): (Male 0.006 ± 3.6, female 0.01 ± 4.5)	
		Biceps (m): male 0.01 ± 4.0, female 0.01 ± 4.1	
		Waist (m): male 0.7 ± 6.4, female 0.7 ± 7.1	
		Hip (m): 0.9 ± 8.0, female 0.9 ± 7.9	
^(33)^	Ansa *et al.*, 2008	NR	Obesity: 1.7
			Overweight: 6.8
^(57)^	Goon *et al.*, 2011	NR	Underweight (WAZ < −2): 43.4
			Stunting (HAZ < −2): 52.7
^(22)^	Adesina *et al.*, 2012	NR	underweight 6.4, overweight 6.3, obesity 1.8 and stunting 5.4
^(61)^	Iyalomhe *et al.*, 2018	NR	Underweight: 24
			Overweight: 3
			Obesity: 1
^(85)^	Omobuwa *et al.*, 2014	NR	Overweight and obesity: 7.6
^(68)^	Ogechi *et al.*, 2007	Weight (Kg): 56.5 ± 8.6(boys), 53.5 ± 6.9 (girls)	Overweight: 4 males and 2 females; Stunting: 67.3 males and 57.8 females
		Height (m): 1.7 ± 6.3 (boys) and 1.6 ± 5.3 (girls)	
		BMI (Kg/m^2^): 20.0 ± 2.6 boys and 20.50 ± 2.38	
^(63)^	Kelvin and Sanusi, 2016	Waist(m): 0.8 ± 0.1	Underweight: 11.7
		Hip circumference(m)0.7 ± 0.1M	Overweight: 8.7
		Waist-hip ratio(m) 0.9 ± 0.1	Obesity: 4.9
		BMI-for- age 45.1 percentile	
^(64)^	Kola-Raji *et al.*, 2017	NR	Stunting: 2.5
			Underweight: 39.3
			Overweight: 8
			Obesity: 0.8
^(97)^	Opara and IEE, 2010	NR	Private school: Underweight: 27.3; Stunting 17.1; Obesity 11.1
			Public school: underweight: 39.4; Stunting: 25.3; Obesity: 0.2
^(52)^	Eze *et al.*, 2017	Height (m): 1.5 ± 10.2	Wasting: 9.3
			Overweight: 6.3
			Obesity: 4.4
		Weight (Kg): 29.7 ± 7.7	Underweight: 0.9
		BMI (Kg/m^2^) 15.7 ± 2.4	Stunting: 0.4
^(43)^	Darling *et al.*, 2020	NR	Underweight:18.6
			Overweight:4.8
			Stunting:15.9
^(84)^	Omigbodun *et al.*, 2010	Males: Height (m) mean (SD): 1.6 (0.13); Weight (Kg): 43.7 (10.61); BMI (Kg/m^2^): 17.31 (2.29)	**Males:** Underweight: 213 (23.1); Height: 213 (23.1); Overweight: 10 (1.1)
		Females: Height (m): 1.6 (0.1); Weight (Kg): 43.3 (9); BMI (Kg/m^2^): 17.9 (2.7)	**Females:** Underweight: 127 (14.5); Height: 69 (7.9); (overweight): 32 (3.7)
^(115)^	Wariri *et al.*, 2020	NR	**Overall**: Thinness:6.8; Overweight/Obesity: 12.4; Stunted: 6.4
			**Gombe:** Overweight/Obesity: 16.1; Thinness: 12
			**Uyo:** Thinness: 5.3; Overweight/Obesity.: 11.3
^(65)^	Lateef *et al.*, 2016	BMI (Kg/m^2^):19.7 ± 2.6	Overweight: 4.7
			Obesity: 0.2
			Underweight: 29.1
			Overweight: male 0.6 and female 6.7
			Obesity: (male 0, female 0.3)
^(69)^	Ogechi *et al.*, 2012	BMI (Kg/m^2^):19.9 ± 2.6 (boys); Girls: 23.0 ± 3.9	NR
^(77)^	Olatona *et al.*, 2020	NR	Undernutrition: 5.4
			Overweight: 10.7
			Obesity: 5.3
^(78)^	Olatona *et al.*, 2022	NR	**Males:** Overweight: 7.1; Obesity: 3.3
			**Females:** Overweight: 7.1; Obesity: 2.8
^(92)^	Onuoha and Eme, 2013	NR	Overweight: 3.0 and 6.7, in males and females, respectively
			Obesity: 1 and 2.5, in males and females, respectively
^(103)^	Samuel *et al.*, 2015	NR	Stunting: 12.1
			Overweight/Obesity: 9.5
^(113)^	Uba *et al.*, 2020	NR	Underweight: 36. Overweight: 9.6
^(15)^	Abdulkarim *et al.*, 2014	Mean BMI 20.31 ± 3.07kg/m^2^, Weight 51.07 ± 10.80 kg, Height 1.6 ± 9.33 m.	Wasting 1.7, Stunting 11.3, Overweight 13.2, Obesity 2.6
^(37)^	Ayogu *et al.*, 2018	NR	Underweight (18.2), Stunting (41.6), Thinness (20.0)
^(50)^	Esimai *et al.*, 2015	mean height 1.55 m mean weight 45.2 kg mean BMI 18.5 kg/m2	Underweight 46.2, Overweight 6.6 Stunting 36.3
^(51)^	Essien *et al.*, 2014	NR	Underweight 27.9 Overweight 7.5
^(58)^	Henry-Unaeze *et al.*, 2011	mean BMI 19.2 ± 3.06kg/m2	Underweight 44.0 Overweight 5.0
^(62)^	Kayode *et al.*, 2020	NR	Underweight 2.2, Overweight 31 Obese 9.3
^(90)^	Oninla *et al.*, 2007	Mean Weight 25.5 ± 6.5 kg. Mean Height of 1.3 ± 12.9m	Underweight 61.2 Wasting 16.8 Stunting 27.6

*Note*: Values are given in percentage unless otherwise indicated.

Table 3 presents findings on food and food group consumption for Nigerian adolescents, from 29 nutritional epidemiology studies that reported consumption data for at least one food and/or food group.^(25–27,31,38–40,56,60,70,71,74,76,77,80,94–96,101,102,104,105,110,113,117–120)^ Starchy staples, encompassing cereals and starchy roots/tubers, emerged as a commonly consumed dietary component, were reported by eleven studies and consumption levels ranged from 28.2% to 96.7%.^(25,26,38,70,74,76,77,96,101,113,117)^ However, only two studies reported consumption levels below 50%.^(25,76)^ Legumes, nuts, and seeds were reported in six studies.^(25,38,60,70,105,117)^ Similarly, consumption of meat, poultry, fish, and seafood ranged from 19.1% to 62.1%, reported in nine studies.^(25,38,60,70,74,76,96,101,117)^ Vegetable consumption was documented across eleven studies with a range from 10.1% to 83.0%, with seven studies indicating proportions below 50%.^(25,38–40,70,71,96,113,117–119)^ Fruit intake ranged from 9.7% to 77.4% and was reported across seventeen studies.^(25,26,31,38,40,60,70,71,74,77,94,96,101,105,117–119)^ Conversely, sweets’ consumption was described in six studies^(26,71,74,94–96)^ showing variation across the studies (range: 35.3–62.4%). Snacking habits were recorded and reported in ten studies (range: 33–96.8%).^(56,60,70,76,77,80,93,96,104,117)^ Additionally, consumption of sugar-sweetened beverages was indicated in 13 studies.^(27,31,40,71,76,94–96,102,110,117,119,120)^ Consumption of such beverages exhibited a wide spectrum, ranging from 8.5% to 99.4%. Only two studies reported egg consumption (range: 3.3–14.3%).^(26,38)^

**Table 3.** Food and food group-based findings

**Food(s) and food group(s)** (proportion of respondents)^a^										Snacking and drinks	
**Author, year**	**Ref.**	**Starchy staples (cereals, starchy roots/ tubers)**	**Legumes, nuts, and seeds**	**Meat, poultry, fish, and seafood**	**Vegetables**	**Fruits**	**Eggs**	**Dairy and milk** **products**	**Sweets**	**Snacking**	**Drinks**
Agofure *et al.*, 2021	^(26)^	47.8 (very often)	NR	NR	NR	38.8 (sometimes)	41.3 sometimes	33.3 (sometimes)	35.3 (sometimes)	NR	NR
Samuel *et al.*, 2021	^(104)^	NR	NR	NR	NR	NR	NR	NR	NR	96.8	NR
Ayogu *et al.*, 2019	^(38)^	96.7	60	46.7	32.1	35.6	13.3	47.8	NR	NR	NR
Okoro *et al.,* 2016	^(74)^	62.3	NR	62.1	NR	67.6	NR	62	62.3^s^	NR	NR
Ikujenlola and Adekoya, 2020	^(60)^	NR	9.5 (≥ 3 times/week), 90.5 (≤ 2 times/week)	49.5 (red meat), 22.4 (white meat), 28.1 (fish), 51.8 (≥ 3 times/week), 48.2 (≤ 2 times/week)	NR	29.1 ≥ 3 times/week; 70.9 ≤ 2 times/week	NR	44.7 (≥ 3 times/week), 55.3 (≤ 2 times/week)	NR	74.9	NR
Orisa andWordu, 2021	^(117)^	54.1 (1-2 times/week)	33.9 (1-2 times/week)	51.3 (fish and meat daily)	47.9 (1-2 times/week)	33.05 (daily)	NR	36.9 (1-2 times/week)	NR	49.6 (daily)	38.1 (1–2 times/week)
Otuneye *et al.*, 2017	^(102)^	NR	NR	NR	NR	NR	NR	NR	NR	NR	35.2
Sanusi *et al.*, 2021	^(105)^	NR	54.6	NR	NR	60 (≤ 1 daily)	NR	25.8	NR	NR	NR
Onyiriuka *et al.*, 2013	^(94)^	NR	NR	NR	NR	11.3 (1–3 times/week)	NR	NR	53.4 (1–3 times/week)	NR	79.4 (1–3 times/week)
Ogunkunle & Oludele, 2013	^(70)^	76.5	52.2	48.80	70.8	69.8	NR	NR	NR	87.2	NR
Ayogu and Nwodo, 2021	^(39)^	NR	NR	NR	29.7 (males), 45.1 (females)	NR	NR	NR	NR	NR	NR
Afolabi *et al.*, 2013	^(25)^	49.3	18.6	84.4	73.7	73.7	NR	73.8	NR	NR	NR
Otemuyiwa and Adewusi, 2012	^(101)^	58 (males), 62 (females)	NR	35 (males),42 (females)	NR	20 (males),40 (females)	NR	10 (males), 25 (females)	NR	NR	NR
Olatona *et al.*, 2018	^(76)^	28.2	NR	32 meat, 10 fish	NR	NR	NR	14	NR	44.0	29, Alcohol: 6
Onyechi &Okolo, 2009	^(118)^	NR	NR	NR	26.7	28.20	NR	NR	NR	33.6	NR
Bamidele *et al.*, 2016	^(40)^	NR	NR	NR	83 (rural), 63.1 (urban)	76.9 (rural), 77.4 (urban)	NR	NR	NR	NR	25.0 (rural), 61.1 (urban)
Olumakaiye *et al.*, 2010	^(80)^	NR	NR	NR	NR	NR	NR	NR	NR	33.00	NR
Oluyinka *et al.*, 2020	^(119)^	NR	NR	NR	10.1	16.10	NR	NR	NR	NR	NR
Agoreyo *et al.*, 2002	^(27)^	NR	NR	NR	NR	NR	NR	22.6	NR	NR	99.4 (beverages except milk), 11.6 (milk beverages), 50.6 (diet soda)
Ansa *et al.*,2008	^(120)^	NR	NR	NR	NR	NR	NR	NR	NR	NR	97.20
Akinola *et al.*, 2022	^(31)^	NR	NR	NR	NR	28	NR	NR	NR	NR	11 (sugar-sweetened beverages daily), 20 (most days of the week)
Funke *et al.*, 2007	^(56)^	NR	NR	NR	NR	NR	NR	NR	NR	51.40	NR
Ogunsile, 2012	^(71)^	NR	NR	NR	10.2	16.4	NR	7.0	50 (sweets), 38.3 (chewing gum)	NR	45.3
Olatona *et al.*, 2020	^(77)^	73.5	NR	NR	NR	9.7	NR	NR	NR	69.6	46.8
Onyiriuka *et al.*, 2013	^(96)^	NR	NR	NR	NR	11.3	NR	NR	53.4 (sugar), 62.4 (ice cream)	76.4	9.40
Onyiriuka etal., 2013	^(95)^	89.6	NR	19.1	15.2	NR	NR	8.1	41.2	NR	58.7
Sholeye *et al.*, 2018	^(110)^	NR	NR	NR	NR	NR	NR	NR	NR	NR	8.5 (carbonated drinks), 44.2 (energy drinks)
Uba *et al.*,2020	^(113)^	78.4	NR	NR	55.2	NR	NR	88.8	NR	NR	NR

a Values are provided in percentage (%), unless otherwise indicated.

Table 4 summarises dietary intake among adolescents in Nigeria. Results show that the amount of protein (range: 27–93.5 g), energy (range: 903–5754.7 kcal) and carbohydrate (range: 82–937.60 g) consumed by majority of adolescents were inadequate^(38,46,68–70)^ also one study reported an inadequate consumption of fat (range: 6.0–157.1 g) by this age group.^(70)^ In contrast, some studies reported an excessive intake of carbohydrate, energy and protein beyond recommended nutrient intake.^(70,82,109,112)^ Only two studies^(30,116)^ reported on fibre intake (range: 13.1–33.1 g) among Nigerian adolescents. One of the studies showed an intake of fibre less than 20 g (when defined as no starch polysaccharide) from foods—or less than 25 g from foods (when defined as total dietary fibre)—which are also the accepted values recommended for the prevention of NCDs.^(122)^ With regards to vitamin intake, the most commonly reported vitamins in the studies included in this systematic review are vitamin A (range: 132.4–9135.2 mg), vitamin C (range: 7.2–80.8 mg), vitamin B_12_ (range: 0–1.4 mg) and folate (range: 0–136.6 mg). Virtually all studies reporting them found insufficient consumption of these vitamins among adolescents.^(30,32,46)^ Information gathered also shows an inadequate intake of minerals and trace elements, particularly calcium, iron, zinc and potassium. There was a report of excessive sodium intake (2225–2404 mg compared to the recommended value of 1200–1500 mg).^(109)^

**Table 4.** Dietary intake by adolescents in Nigeria

Author, year (ref.)	Location	Subject	Method of assessment	Intake reported	Intake value		Nutritional targets		Key findings
Ayogu, 2019 ^(38)^	Ede- Oballa, Southeast	450 in-school children and adolescents	DDS	Energy (Kcal)	Male	Female	Males	Females	Energy, protein and carbohydrate intakes did not meet the recommended nutrients intake for neither sex. However, the girls’ mean carbohydrate intake was higher than boys.
					Data for 10–12 years school adolescents				
					1775.6 ± 508.8	1740.1 ± 452.6	260	2350	
				Protein (g)	45.1 ± 14.7	48.5 ± 24.0	47	58	
				Fat (g)	54.1 ± 25.2	60.1 32.6	NA	NA	
				Carbohydrate (g)	274.4 ± 96.1	284.1 ± 164.1	1430	1293	
					Data for 10–12 years school adolescents				
				Energy (Kcal)	1917.8 ± 559.3	1771.1 ± 681.8	2900	2490	
				Protein (g)	40.2 ± 26.6	35.9 ± 23.2	45	49	
				Fat (g)	51.9 ± 35.9	75.9 ± 44.6	NA	NA	
				Carbohydrate (g)	154 ± 94.5	321.3 ± 304.9	1595	1370	
Sanusi et al, 2021 ^(105)^	Ibadan	433 school-aged adolescents	24- hour dietary recall	Prudent Pattern (n = 474)					Foods consumed by the target population does not provide an adequate supply of nutrients
				Energy (kcal)	1592.8 ± 578		NR	NR	
				Protein (g)	55.2 ± 22.7		NR	NR	
				Carbohydrates (g)	259 ± 96.7		NR	NR	
				Traditional South-Western Nigerian Pattern (n = 481)					
				Energy (kcal)	1414.7 ± 494.5		NR	NR	
				Protein (g)	43.2 ± 18.4		NR	NR	
				Carbohydrates (g)	235.8 ± 88.3		NR	NR	
Ogunkunle and Oludele, 2013 ^(70)^	Ila Orangun, southwest	302 public school adolescents	FFQ	Energy (kcal)	Below reference (BR): 24.4; Above reference (AR): 200		NR	NR	Insufficient calcium and iron intake were observed.
				Carbohydrates (g)	BR 43; AR 187		NR	NR	
				Lipids (g)	BR 9; AR 153		NR	NR	
				Proteins (g)	BR 127;AR 73		NR	NR	
				Calcium (mg)	BR 281; AR 21		NR	NR	
				Iron (mg)	BR 145; AR 157		NR	NR	
Onimawo IA *et al.*, 2010 ^(121)^	Abia State	249 rural school children	24-hour dietary recall and FFQ	Iron (mg)	6.8		10		According to the findings of this study, children’s iron intake was approximately 30% lower than RDA.
				Protein (g)	32		34		
				Fat (g)	32		NR		
				Carbohydrate (g)	250		NR		
				Energy (kcal)	1220		1800–2000		
				Zinc (mg)	4.5		10		
Anyika *et al.*, 2009 ^(34)^	Abia State	160 Secondary school and university students	Questionnaire	Energy (kcal)	3838.3 for secondary school students (SS); 5754.7 for university students (US)		1957.4–2158.2		The conclusion of this study indicate that energy intake was higher than the recommended nutrient intake in all the schools studied.
				Carbohydrate(g)	SS: 757.1; US: 937.60		NR	NR	
				Protein (g)	SS: 93.5; US: 135.4		NR	NR	
				Fat (g)	SS: 50; US: 157.1		NR	NR	
				Iron (mg)	SS: 31.6; US: 37.1		NR	NR	
				Vit. A (µg RE)	SS: 6296.1; US: 9135.2		NR	NR	
				Vit C. (mg)	SS: 94.1; US: 179.7		NR	NR	
Akinyemi& Ibraheem, 2009 ^(32)^	Lagos	40 secondary-school adolescents	Questionnaire	Energy (kcal)	1546.8		2310–2350	2600–3070	This study shows inadequate nutrient intake.
				Protein (g)	56		29–30	30–38	
				Vitamin C (mg)	30.3		20–30	20–30	
Ene-Obong *et al.*, 2003 ^(46)^	Nsukka, Enugu state	135 in-school adolescents	3-day weighted food intake	Vitamin A (ug RE/day)					Vitamin C intake was below recommendations, while vitamin A exceeded recommendations
				13–15 years	820 ± 61	766 ± 26	137	131	
				16–18 years	765 ± 20	776 ± 25	127	126	
				19–20 years	797 ± 19	NR	133	NR	
				Vitamin C (mg/day)					
				13–15 years	24.3 ± 1.2	19.5 ± 5.1	81	65	
				16–19 years	23.7 ± 0.7	15.3 ± 2.8	77	51	
				19–20 years	27.3 ± 8.0	NR	91	NR	
Otemuyiwa and Adewusi, 2012 ^(101)^	Osun and Ondo State	402 university students	FFQ	Obafemi Awolowo University					70% of participants met RDA for iron, whereas just 15% met the calcium RDA. Iron, calcium and phosphorus intake did not differ significantly between sexes or institutions.
				Protein (g)	84 ± 16	83 ± 25	1.1	NR	
				Iron (mg)	15 ± 4b	13 ± 5	10	15	
				Calcium (mg)	311 ± 110	319 ± 203	800		
				Phosphorus (mg)	771 ± 219	795 ± 323	1000		
				Adekunle Ajasin University					
				Protein (g)	83 ± 14	88 ± 22	1.1		
				Iron (mg)	15 ± 4	14 ± 4	10	15	
				Calcium (mg)	343 ± 18	369 ± 250	800		
				Phosphorus (mg)	724 ± 292	849 ± 333	1000		
Shokunbi& Ukangwa, 2021 ^(109)^	Ilishan- Remo, Ogun State	488 secondary school adolescents	Questionnaire	Sodium (mg)	2404 ± 902	2225 ± 971	1200–1500		Sodium intake exceeded RDA, while potassium fell below it.
				Potassium (mg)	1384 ± 972	1298 ± 588	2300–3000		
Tassy *et al.*, 2021 ^(112)^	Ibadan, Oyo state	955 secondary-school adolescents	Multiple-pass 24h dietary recall	Energy (kcal)	1590 ± 295		NR	NR	According to findings of study, there were greater micro-nutrients deficiencies among adolescents.
				Carbohydrates (g)	271.1 ± 50		NR	NR	
				Protein (g)	50 ± 15		NR	NR	
				Calcium (mg)	301 ± 68	1100	NR	NR	
				Iron (mg)	11 ± 2	16.4	NR	NR	
Oguntona and Kanye, 1995 ^(72)^	Ogun state	187 secondary-school adolescents	Questionnaires and 24-hour recall	Energy (kcal)	2593.2 ± 239		NR	NR	This study showed that protein intake was relatively high for both sexes, with 10% of total protein intake coming from animal sources.
				Protein (g)	62.6 ± 12.3		NR	NR	
				Animal protein (g)	4.1 ± 1.4		NR	NR	
				Oils and fats (g)	29.8 ± 0.6		NR	NR	
				Carbohydrates (g)	82 ± 12.4		NR	NR	
				Cholesterol (g)	9.1 ± 0.2		NR	NR	
				Fibre (g)	33.1 ± 0.5		NR	NR	
Akinlade *et al.*, 2014 ^(30)^	Oyo state	821 Secondary School adolescents	Questionnaire	Calorie (kcal)	1726.8 ± 327.5		1040 – 2197		Carbohydrate intake was above RNI for both sexes, while fat, iron and zinc intakes were within RNI. Protein, vitamin A, C and B12, folate, and calcium were below recommendations.
				Protein (g)	39.3 ± 10.7		11 – 57.8		
				Carbohydrate(g)	170.6 ± 56.5		89.8 – 294		
				Fibre (g)	13.1 ± 10.7		0 – 68.9		
				Fat (g)	29.9 ± 14.1		1.2 – 54.2		
				Vitamin A (RE)	478.4 ± 293.7		19.6 – 978.9		
				Vitamin C (mg)	7.2 ± 5.7		0– 89.9		
				Folate (mcg)	136.6 ± 118.1		0– 642.1		
				Vitamin B12 (mcg)	1.4 ± 1.1		0 – 6.4		
				Calcium (mg)	305.3 ± 216.2		27– 885.7		
				Zinc (mg)	6.9 ± 3		0.01 – 15.9		
				Iron(mg)	11.3 ± 4.7		3.2 – 21.1		
Ogechi *et al.*, 2007 ^(68)^	Umuahia, Abia state	190 secondary schools Adolescents	7 d weighed food inventory	Energy (kcal)	2683.1 ± 113.9		NR	NR	Males consumed significantly more carbohydrates, fats and energy.
				Protein (g)	54.4 ± 7.9		NR	NR	
				Fat (g)	43.7 ± 3.8		NR	NR	
				Carbohydrate(g)	518.9 ± 27.7		NR	NR	
Ogechi, 2012 ^(69)^	Umuahia, Abia state	416 secondary School adolescents	7 d weighed food inventory	Protein (g)	53.9 ± 7.2	50.9 ± 9.3	NR	NR	Boys had higher energy intake but still below recommended levels for both sexes
				Fat (g)	43.7 ± 1.9	47.4 ± 9.9	NR	NR	
				CHO (g)	517.3 ± 30.1	426.6 ± 27.5	NR	NR	
				Energy (kcal)	2673.7 ± 12.8	2343.5 ± 10.9	NR	NR	
Oranusi *et al.*, 2007 ^(98)^	Zaria, Kaduna state	44 members (5 families)	6 d food inventory	Energy (kcal)	1194 ± 114.7	1062.6 ± 98.7	NR	NR	Energy intake was below RDA.
Erinoso *et al.*, 1992 ^(49)^	Olodo, Oyo State	400	Questionnaire	Energy (kcal)	903 ± 134		36 ± 6		Results indicate a significant deviation from the established reference values and poor nutritional status.
				Protein (g)	27 ± 7		89 ± 24		
				Iron (mg)	13 ± 7		10 6 ± 75		
				Calcium (g)	128 ± 71		22 ± 17		
				Riboflavin (mg)	1 ± 0.4		72 ± 28		
				Vitamin C (mg)	12 ± 5		55 ± 20		
Onabanjo and Balogun, 2014 ^(88)^	Ogun State	127	Questionnaire	Energy (kcal)	2028.9 ± 228.7	1738.9 ± 216.7	NR	NR	The majority (59.8%) of adolescents had iron intake below the recommended nutrient intake.
				Carbohydrates	276.8 ± 15.6	251.3 ± 10.6	NR	NR	
				Fats (g)	47.7 ± 3.8	40.3 ± 2.6	NR	NR	
				Zinc (mg)	10.3 ± 1.8	8.6 ± 1.2	NR	NR	
				Phosphorus (mg)	771.3 ± 106.3	616.2 ± 101.6	NR	NR	
				Niacin (mg)	3.7 ± 0.6	2.5 ± 0.5	NR	NR	
				Vitamin C (mg)	80.8 ± 16.4	72.6 ± 20.1	NR	NR	
Yunusa *et al.*, 2014 ^(116)^	Nigeria	270	Questionnaire	Energy (kcal)	2014.4 ± 194.6	1441.1 ± 169.3	2036.3	2036.3	Overall energy intake was higher among males, compared to females.
				Water	36.3 ± 11.8	501.7 ± 72.5	2800	2450	
				Protein (g)	48.3 ± 9.6	36 ± 9.6	60.1	60.1	
				Fat (g)	24.5 ± 7.3	6 ± 2.9	69.1	69.1	
				Carbohydrate (g)	393.7 ± 26.1	305.4 ± 11.2	290.7	290.7	
				Dietary fibre (g)	23.5 ± 8.2	18.9 ± 2.4	30	30	
				PUFA (g)	10.8 ± 2.8	1.8 ± 0.6	10.0	10	
				Vitamin A (mg)	132.4 ± 23.1	158.9 ± 29.6	900	1100	
				Total folic acid (mg)	70.3 ± 21.5	50.7 ± 6.8	400	400	

*Note*: RNI, Recommended Nutrient Intake; NR, Not reported; RDA, Recommended Dietary Allowance; PUFA, Polyunsaturated fatty acids; FFQ, Food Frequency Questionnaire; DDS, Dietary Diversity Score.

Table 5 presents a summary of dietary habits among Nigerian adolescents. The studies included in the table report findings related to key dietary characteristics such as the number of meals, observing breakfast, eating lunch, eating dinner, and skipping meals. Specifically, the table includes findings from twenty-one studies.^(17,25,26,31,51,60,61,70,71,76,80,86,94,95,102,104,105,113,117,118,123)^ Among these studies, nine^(26,60,61,71,76,80,86,105,117)^ reported the number of meals based on a three-square meal basis. Thirteen^(26,31,60,70,71,102,105,118)^ studies provided information on the proportion of participants who observe breakfast, while seven^(70,86,94,102,105,113,118)^ studies reported the proportion of participants who observed lunch meal. Only one^(102)^ study reported the responses of participants regarding the order of importance of meals, fasting to lose weight, using diet pills to lose weight, infrequent intake of fruits/vegetables, consuming alcohol and smoking cigarettes. Furthermore, eight^(17,25,51,60,76,94,104,117)^ studies examined fast-food consumption, and four^(26,61,95,102)^ studies investigated factors that motivate dietary intake.

**Table 5.** Findings on dietary habits of the available studies

Ref.	Author, year	Primary aim of the study	Findings
^(26)^	Agofure *et al.*, 2021	This study investigated the dietary patterns and nutritional status of the female adolescents in Amai Secondary Commercial School, Delta State, Nigeria.	- Proportion of meals (three-square meals): 34.4% of participants reported having meals sometimes. - Proportion of participants observing breakfast: 38.3% observed breakfast very often. - Proportion of participants eating fruit after meals: 48.3% ate fruit sometimes. - Proportion of participants skipping meals: 41.8% skipped meals sometimes. - Factors motivating dietary intake: Nutritional values (35.3%), taste (26.9%). - Order of importance: Breakfast (24.40%), Lunch (21.9%), Dinner (14.4%).
^(104)^	Samuel *et al.*, 2021	Study explored adolescents’ dietary patterns (DP) and nutritional status focusing on out-of-school adolescents	- Proportion of participants eating home-cooked meals: 90.5%. - Proportion of participants eating fast food: 87.8%.
^(105)^	Sanusi *et al.*, 2021	This study examined the contribution of food to nutrient intake, meal, and dietary patterns among children aged 9–13 years in Ibadan, Nigeria	- Proportion of meals (three-square meals): 85%. - Proportion of participants observing breakfast: 95%. - Proportion of participants eating lunch: 85%. - Proportion of participants eating mid-morning meals: 48%. - Proportion of participants eating dinner: 85%.
^(60)^	Ikujenlola and Adekoya, 2020	This study examined the dietary habits, nutritional status, and socio-demographic characteristics of female undergraduates in selected public and private universities in Osun State, Southwestern Nigeria	- Proportion of meals (three-square meals): 33% of participants reported having meals. - Proportion of participants observing breakfast: 52.4% observed breakfast. - Proportion of participants skipping meals: 86%. - Proportion of participants eating food made outside home: 10.5%. - Proportion of participants eating fast food: 16.5%. - Proportion of participants eating home-cooked meals: 73%. - Proportion of participants eating from school cafeteria: 54.8%.
^(117)^	Orisa and Wordu, 2021	This study was designed to assess the diet, physical activity, and food consumption patterns of adolescent girls in Port Harcourt, Rivers State	- Proportion of meals (three-square meals): 61.43% of participants reported having meals. - Proportion of participants skipping meals: 55.71% skipped breakfast sometimes. - Proportion of participants eating fast food: 55.71% ate fast food occasionally.
^(102)^	Otuneye *et al.*, 2017	To determine the dietary eating patterns and nutritional status among adolescents in secondary schools within Abuja Municipal area council	- Proportion of participants eating breakfast every day: 4.6%. - Proportion of participants eating lunch: 15%. - Proportion of participants eating dinner: 16.6%. - Proportion of participants fasting to lose weight: 16.3%. - Proportion of participants inducing vomiting/taking laxatives to lose weight: 5%. - Proportion of participants using diet pills to lose weight: 9%. - Proportion of participants eating less food or fat to lose weight: 28%. - Factors motivating dietary intake: Taste good (35.2%), balanced diet (34.2%), satisfying (15.6%), don’t know (15%), good nutritional knowledge (34.8%).
^(70)^	Ogunkunle and Oludele, 2013	This study was designed to assess the food intake and describe the meal pattern of adolescents attending public secondary schools in Ila Orangun, southwest Nigeria	- Proportion of participants observing breakfast: 62.2%. - Proportion of participants eating lunch: 6.9%. - Proportion of participants eating dinner: 95.1%.
^(118)^	Onyechi and Okolo, 2009	The objective of this study is to determine the prevalence of obesity among undergraduates living in halls of residence in University of Nigeria Nsukka campus and to obtain information on their feeding pattern, physical activity, and health status	- Proportion of participants observing breakfast: 64.9%. - Proportion of participants eating food made outside home: 40.6%. - Proportion of participants eating home-cooked meals: 46.6%. - Proportion of participants eating lunch: 62.6%. - Proportion of participants eating dinner: 64.1%.
^(80)^	Olumakaiye *et al.*, 2010	Association between nutritional status of adolescents and food consumption pattern	- Proportion of meals (three-square meals): 66.1%.
^(31)^	Akinola *et al.*, 2022	To describe dietary habits, physical activity, and sleep patterns among secondary-school adolescents	- Proportion of participants observing breakfast: 68.9% (3–5 d/week). - Proportion of participants skipping meals: 10%.
^(61)^	Iyalomhe *et al.*, 2018	This study aimed to optimise the health needs of Nigerian adolescents by determining the dietary habits and the nutritional status of adolescents using anthropometry	- Proportion of meals (three-square meals): 69.2%. - Proportion of participants skipping meals: 39.2%. - Factors influencing dietary intake: Parental influence (87%), taste of food (71%), mass media reports (61%), and culture (55%).
^(71)^	Ogunsile, 2012	The main objective of this study was to determine the effects of dietary patterns and body mass index on the academic performance of in-school adolescents in Ekiti State	- Proportion of meals (three-square meals): 14.1%. - Proportion of participants observing breakfast: 16.4%.
^(123)^	Olatona *et al.*, 2022	The objective of this study was to determine the relationship between breakfast skipping and the prevalence of obesity among secondary-school adolescents in Lagos State	- Proportion of participants observing breakfast: 57.4%. - Proportion of participants aged 16-19 years having breakfast: 52.2%. - Proportion of participants aged 13–15 years having breakfast: 43%. - Proportion of participants aged <13 having breakfast: 34.7%.
^(86)^	Omuemu and Oko-Oboh, 2015	This study aimed to determine the pattern of meal consumption among in-school adolescents in Benin City	- Proportion of meals (three-square meals): 71.6%. - Proportion of participants observing breakfast: 85.9%. - Proportion of participants eating lunch: 90.2%. - Proportion of participants eating dinner: 95.5%.
^(95)^	Onyiriuka *et al.*, 2013	To describe the pattern of snack consumption among adolescent Nigerian urban secondary schoolgirls	- Proportion of participants eating snacks between meals: 74.8%. - Factors motivating dietary intake: Taste (88.2%).
^(94)^	Onyiriuka *et al.*, 2013	To describe the eating habits of adolescent urban secondary-school girls in Benin City, Nigeria	- Proportion of participants observing breakfast: 53.7%. - Proportion of participants skipping meals: 48%. - Proportion of participants having breakfast: 46 (breakfast), 22 (dinner). - Proportion of participants eating fast food: 60 (with soft drinks) and 76 (without soft drinks). - Proportion of participants eating lunch: 69.6%. - Proportion of participants eating dinner: 78.5%.
^(17)^	Adebimpe, 2019	The objective of this study was to determine the prevalence and knowledge of risk factors of childhood obesity among school-going children in Osogbo, south-western Nigeria	- Proportion of participants eating fast food: 53.1%. - Proportion of participants taking snacks between meals: 86.7%. - Proportion of participants often taking sweets: 75.6%. - Proportion of participants eating ice cream: 74.8%. - Proportion of participants often taking soft drinks: 87.9%. - Proportion of participants having good nutritional knowledge: 91.9%. - Proportion of participants having low self-esteem: 61.7%. - Proportion of participants taking alcohol: 2.1%. - Proportion of participants smoking cigarettes: 0.8%.
^(25)^	Afolabi *et al.*, 2013	The aim of the study was to assess the pattern of consumption and the contribution of fast foods to nutrient intake of undergraduates of the Federal University of Agriculture Abeokuta (FUNAAB)	- Proportion of participants eating fast food: 60% (males), 40% (females). - Proportion of participants eating fast food: 53.6% (males), 35.0% (females).
^(51)^	Essien *et al.*, 2014	The aim of this study was to determine the nutrition knowledge and nutritional status of children attending a secondary school in Sokoto metropolis	- Proportion of participants eating fast food: 68.8%. - Proportion of participants taking snacks between meals: 82.1%. - Proportion of meals (three-square meals)^(51)^: 3.3% (once), 13.3% (twice), 56.3% (thrice), 27.1% (more than thrice).
^(76)^	Olatona *et al.*, 2018	This study aimed to assess the dietary pattern and metabolic risk factors of non-communicable diseases among university undergraduate students in Lagos State	- Proportion of meals (three-square meals): 7% (once), 57.1% (twice), 31% (thrice), 5% (more than thrice). - Proportion of participants taking snacks between meals: 44%. - Proportion of participants eating fast food: 22.7% (daily), 18.9% (4–6 times/week), 58.4% (≤3 times/week). - Proportion of participants often taking soft drinks: 29.0%. - Proportion of participants having good nutritional knowledge: 22.9%. - Proportion of participants observing breakfast: 22.5%.
^(113)^	Uba *et al.*, 2020	This study aimed to investigate the nutritional status of adolescent girls in a selected secondary school in Nigeria	- Proportion of participants observing breakfast: 91.6%. - Proportion of participants eating lunch: 84.8%.

Table 5 provides information on studies reporting dietary habits of Nigerian adolescents. With regards to meal patterns the proportion of participants reporting consuming three-square meals varied across studies, ranging from 33% to 85%. A majority of participants generally observed breakfast, with proportions ranging from 16.4% to 95%. Lunch and dinner were also commonly consumed meals, with proportions ranging from 6.9% to 95.5%. Skipping meals was prevalent among adolescents, with proportions ranging from 10% to 86%. Breakfast skipping was particularly common, reported by 48% to 86% of participants in different studies. Fast food consumption was widespread among adolescents, with proportions ranging from 16.5% to 87.8%. Some studies highlighted a higher prevalence of fast-food consumption among females. Fruit consumption varied across studies, with proportions ranging from 48.3% to not been reported. The frequency of fruit consumption was generally moderate, with a significant proportion reporting eating fruit sometimes.

Fig. 2 shows a summary of results from studies reporting anthropometric indicators and these studies have been ordered by year, to provide a trend over time. For all 5 indicators (obesity, stunting, thinness, underweight and wasting) the trends appear to decrease over time.

## Discussion

To the best of our knowledge, this systematic review represents the most comprehensive observational analysis on dietary profile and nutritional status of adolescents in Nigeria. We reviewed and synthesised results from a total of 102 nutritional epidemiological studies among Nigerian adolescents (10–19 years) with a total population of 67,844 participants. However, only 13 % of the included studies were classified being at low risk of bias in terms of methodological quality and with the majority published in unindexed journals (89.2%). No prospective studies were found on the topic. Since included studies were cross-sectional, their reliance on self-reported, unvalidated tools for dietary assessment or anthropometric classification was common. The majority (i.e. 73.5%) of the surveys took place within education settings (e.g. school or university) and some studies focused only on males (12/102), but no study focusing only on females was found. The rest had both males and females. From the results, it is evident that a significant amount of nutrition research has been conducted involving Nigerian adolescents, but there is large variability with regards to assessment and quality of methods, with a substantial proportion of studies using unvalidated tools and instruments and methodologies with high risk of bias.

Although there were no studies focusing exclusively on male participants, some of the nutritional issues reported among the included studies were related mostly to undernutrition, particularly among adolescent girls. Such findings are in line with a UNICEF report, that reported a significant increase in undernourishment among adolescent girls in Nigeria, with the number rising from 5.6 million in 2018 to 7.3 million in 2021.^(124)^ According to the same data, there are evident malnutrition challenges among school-aged adolescents, with a prevalence of thinness and overweight, 10% and 8% respectively. Further, among girls aged 18 and above, 10% are underweight, while 33% are overweight. The prevalence of anaemia among women aged 15–49 years is 55%. Additionally, 55% of households in Nigeria consume salt with iodine, which is an important indicator for addressing iodine deficiency disorders.

In addition, results from the National Nutrition and Health Survey 2018 show that the prevalence of acute malnutrition was more than four times higher for adolescents (15–19 years) than adult women (20–49 years), 19 per cent compared to 4 per cent, respectively.^(125)^ This report underscored the urgency of developing effective interventions to improve the nutrition of adolescent girls, as they play a crucial role in birth outcomes and subsequent nutrition throughout the lifecycle. Improving nutrition in adolescent girls is critical to improving the nutrition status of the entire population.

Nigeria is one of the 12 countries hit hardest by the global food and nutrition crisis, which has been exacerbated by factors such as COVID-19, conflict, and drought. The dietary diversity of adolescent girls’ and women’s diets is too low, particularly in rural areas and poor households.^(126)^ A recent situation report from the UN Office for the Coordination of Humanitarian Affairs indicates a nutrition crisis is occurring in 6 regions.^(127)^ According to the same report the number of adolescents with severe acute malnutrition requiring inpatient care between January and April 2023 increased by 61% compared to the same period in 2022. Thinness trends in our study are similar to data from UNICEF, showing a temporal downward trend.^(128)^ According to the 2023 edition of the Joint Child Malnutrition Estimates by UNICEF/WHO/World Bank Group that in 2012, Nigeria had a stunting prevalence rate of 37.7%, indicating a high level of stunting. However, by 2022, this rate decreased to 34.2%.^(129)^ In the same report, Nigeria had an overweight prevalence rate of 2.5% in 2012, which remained relatively stable at 2.2% in 2022. Wasting for 2020 was 6.5 % and considered a “medium” prevalence threshold, i.e. 5–10%. It is important to note that the data provided is limited to specific years (2012, 2020, and 2022), does not include most recent estimates of all indicators and reflect mostly the children’s population, not adolescents.

In terms of global targets for nutrition for 2025 outlined in the United Nations Sustainable Development Goals Agenda 2030,^(130)^ Nigeria appears to have made slight progress. This limited progress is also reflected in the updated, Global Nutrition Report 2021^(131)^ that shows the country being “*on course”* to fulfil one of the global nutrition objectives for which there is adequate data to assess progress. These goals and global programmes have focused mostly on nutrition in childhood and adults, but data on adolescents appear to have been overlooked. National and global policy has also overlooked adolescent and youth nutrition and the UN Decade of Action on Nutrition (2016–25) has no adolescent or youth-specific nutrition indicators. With the current global challenges, malnutrition in all its forms may worsen. Kidnapping, communal conflict, inflation, urbanisation and banditry may have impeded Nigeria’s capacity to make progress.^(132)^ A review in 2020^(133)^ reported that Nigeria’s stunting rate is 37%, making it the world’s second-most-stunted affected nation.

A previous review,^(134)^ in line with ours, pointed out challenges on insufficiency and scarcity of the data on nutritional status. This is consistent with many low and middle-income countries where malnutrition has been a major concern and, in our synthesis, the included studies reported a range of malnutrition issues, starting from underweight and stunting to overweight and obesity. This is a strong signal reflecting the presence of a double burden of malnutrition. The argument is further supported by a recent analysis on temporal trends in overweight and obesity in Nigerian adolescents and young adults,^(135)^ that reported the co-existence of under- and over-nutrition challenges.

Furthermore, the included studies in our review that reported intake across different regions in Nigeria, consistently showed inadequate intake of certain nutrients. In particular, energy, protein, iron and calcium intake were the most reported inadequacies and, in some cases, certain nutrient intakes exceeded established recommendations. Taken together, these findings suggest that current or future efforts targeting adolescent nutrition in Nigeria should consider a region- and context-specific approach to address the identified dietary gaps.

Our findings also illustrate an adolescent population with consumption patterns varying widely, but with an overall picture that indicates frequent and widespread consumption of starchy staples, sugar-sweetened beverages and snacks and a highly variable intake across studies of other essential food groups like vegetables, fruits and dairy products. This is in line with a review on dietary intake of schoolchildren and adolescents in developing countries,^(136)^ where it was reported that in Nigeria this population group had inadequate consumption of vegetables, micro-nutrients, fruits and animal protein. In the same analysis, a significant increase in the consumption of snacks and energy-dense nutrient-poor foods and drinks was reported. Another recent analysis also showed that adolescents, in Ogun state (south-western Nigeria), primarily consumed starchy foods, with limited dietary diversity.^(100)^

In 2021^(6)^ a position paper from the Nutrition Society of Nigeria called for urgent action“… *to bridge the identified policy and data gaps, enhance coordination and increase delivery platforms to reach adolescents with a minimum package of nutrition interventions giving special consideration for nutritional needs of pregnant adolescent mothers.”* Although adolescents have increasing nutritional requirements and constitute about 21% (more than 41 million) of the Nigerian population, surveillance of their well-being and nutrition remains largely underestimated, inconsistently measured and not prioritised for nutrition interventions. Considering all complexities, efforts to address this situation must be culturally relevant, region-specific and address the identified challenges. Considering earlier findings that highlight the influence of the food environment on adolescents’ food choices, it’s evident that fast-food establishments offering processed foods rich in fat, salt and sugar are gaining popularity in Nigeria.^(137)^ This trend is especially pronounced among adolescents. Future studies should dissect adolescents’ autonomy and agency within the food environment in Nigeria. Irrespective of context, adolescents have a lot to say about why they eat what they eat, and insights into factors that might motivate them to change. Efforts to improve food environments and ultimately adolescent food choice should harness widely shared adolescent values and input beyond nutrition or health.

### Limitations

Although our report is the most comprehensive review of adolescent nutrition in Nigeria, concerns about the scarcity of studies and poor methodological rigour undermine establishment of strong inferences. The available observational studies on adolescent nutrition reveal several methodological limitations, including issues with study design, confounder control, statistical analysis, and sampling, highlighting a crucial need for enhanced investment in robust and rigorous nutrition research to better understand and support adolescent health. In addition, further research is currently ongoing and we did not include intervention studies, but based on current findings and in order to address the identified research gaps, we are conducting an epidemiologic study, for which we have obtained approval from the Yobe State Ministry of Health and Human Service (YB/MOH/HREC/04/22/008). More precise evidence to understand the key nutritional challenges and context of food choices of Nigerian adolescents is needed to increase the potential for impactful and tailored actions.

## Conclusions

Our review on the state of nutrition of Nigerian adolescents showcases both the inherent strengths and limitations of nutrition research in the country, emphasizing the urgent need for targeted, evidence-based interventions to address the double burden of malnutrition. This is further nuanced due to cultural and regional differences and other socio-cultural determinants. Overall, findings underscore the need for more rigorous research and establishment of nutrition surveillance for malnutrition in all its forms among Nigerian adolescents.

## Supporting information

Gabriel et al. supplementary material Gabriel et al. supplementary material

## Abbreviations

**BMI:** Body mass index; **DDS:** Dietary diversity score; **FFQ:** Food frequency questionnaire; **HAZ:** Height-for-age z-score; **JBI:** Joanna Briggs institute; **LMICs:** Low- and middle-income countries; **NCD:** Noncommunicable diseases; **NR:** Not reported; **PRISMA:** Preferred reporting items for systematic reviews and meta-analyses; **PROSPERO:** International prospective register of systematic reviews; **PUFA:** Polyunsaturated fatty acids; **RDA:** Recommended dietary allowance; **RNI:** Recommended nutrient intake; **SDGs:** Sustainable Development Goals; **UN:** United Nations; **UNICEF:** United Nations Children’s Fund; **WAZ:** Weight-for-age *z*-score; **WHO:** World Health Organization

## Supplementary material

The supplementary material for this article can be found at https://doi.org/10.1017/jns.2024.34

## References

[1] Department of Economic and Social Affairs Population Division. World Population Prospects 2022. New York: UNDESA; 2022.

[2] Inter-Agency & Expert Group on Sustainable Development Goal Indicators. Annex III: Revised list of Global Sustainable Development Goal Indicators. New York, NY, USA: United Nations Statistics Division; 2017.

[3] Norris SA , Frongillo EA , Black MM , et al. Nutrition in adolescent growth and development. Lancet. 2022;399:172–184. 34856190 10.1016/S0140-6736(21)01590-7

[4] Afshin ASP , Fay KA , Cornaby L , et al. Health effects of dietary risks in 195 countries, 1990–2017: a systematic analysis for the global burden of disease study 2017. The Lancet. 2019;393:1958–1972. 10.1016/S0140-6736(19)30041-8 PMC6899507 30954305

[5] Twig G , Yaniv G , Levine H , et al. Body-mass index in 2.3 million adolescents and cardiovascular death in adulthood. N Engl J Med 2016;374:2430–2440. 27074389 10.1056/NEJMoa1503840

[6] Samuel F. Securing the future of Nigerian adolescents through nutrition: a position paper of the nutrition society of Nigeria (NSN). Niger J Nutr Sciences. 2021;42:1.

[7] Development Initiatives. 2022 Global Nutrition Report: Stronger Commitments for Greater Action. Bristol, UK: The Global Nutrition Report; 2022.

[8] Osendarp S , Verburg G , Bhutta Z , et al. Act now before Ukraine war plunges millions into malnutrition. Nature. 2022;604:620–624. 35449463 10.1038/d41586-022-01076-5

[9] Muka T , Glisic M , Milic J , et al. A 24-step guide on how to design, conduct, and successfully publish a systematic review and meta-analysis in medical research. Eur J Epidemiology. 2020;35:49–60. 10.1007/s10654-019-00576-5 31720912

[10] Glisic M , Raguindin PF , Gemperli A , et al. A 7-step guideline for qualitative synthesis and meta-analysis of observational studies in health sciences. Public Health Reviews. 2023;44:1605454. 37260612 10.3389/phrs.2023.1605454 PMC10227668

[11] Moher D , Liberati A , Tetzlaff J , et al. Preferred reporting items for systematic reviews and meta-analyses: the PRISMA statement. PLoS Med 2009;6:e1000097. 19621072 10.1371/journal.pmed.1000097 PMC2707599

[12] Sawyer SM , Azzopardi PS , Wickremarathne D , et al. The age of adolescence. Lancet Child Adolesc Health. 2018;2:223–228. 30169257 10.1016/S2352-4642(18)30022-1

[13] Briggs J. Checklist for Analytical Cross Sectional Studies. Adelaide: The Joanna Briggs Institute; 2017.

[14] Page MJ , McKenzie JE , Bossuyt PM , et al. The PRISMA 2020 statement: an updated guideline for reporting systematic reviews. BMJ 2021;372:n71. 33782057 10.1136/bmj.n71 PMC8005924

[15] Abdulkarim AA , Otuneye AT , Ahmed P , et al. Adolescent malnutrition: prevalence and pattern in Abuja municipal area council, Nigeria. Niger J Paediatrics. 2014;41:99–103.

[16] Abidoye RO , Akande PA. Nutritional status of public primary school children: a comparison between an upland and riverine area of Ojo LGA, Lagos state Nigeria. Nutr Health. 2000;14:225–240. 11142611 10.1177/026010600001400404

[17] Adebimpe WO. Prevalence and knowledge of risk factors of childhood obesity among school-going children in Osogbo, south-western Nigeria. Malawi Med J. 2019;31:19–24. 31143392 10.4314/mmj.v31i1.4 PMC6526345

[18] Adeomi AA , Adelusi IO , Adedeji PO , et al. Nutritional status and cardiometabolic health among adolescents; findings from southwestern Nigeria. BMC Nutrition. 2019;5:45. 32153958 10.1186/s40795-019-0308-5 PMC7050742

[19] Adeomi AA , Olodu MD , Akande RO , et al. Adolescent obesity and its association with socio-demographic profile, lifestyle factors, dietary and physical activity patterns; findings from southwestern Nigeria. West Afr J Medicine. 2022;39:119–126. 35277954

[20] Adeomi AA , Fatusi A , Klipstein-Grobusch K. Food security, dietary diversity, dietary patterns and the double burden of malnutrition among school-aged children and adolescents in two Nigerian states. Nutrients. 2022;14:789. 35215439 10.3390/nu14040789 PMC8875779

[21] Adeomi AA , Fatusi A , Klipstein-Grobusch K. Individual and contextual factors associated with under- and over-nutrition among school-aged children and adolescents in two Nigerian states: a multi-level analysis. Public Health Nutr 2022;25:2339–2351. 10.1017/S1368980022000258 PMC9991795 35067272

[22] Adesina AF , Peterside O , Anochie I , et al. Weight status of adolescents in secondary schools in port Harcourt using body mass index (BMI). Ital J Pediatrics. 2012;38:1–7. 10.1186/1824-7288-38-31 PMC3412731 22823927

[23] Adinma J , Emeka EA , Egeonu RO , et al. Dietary intake and nutritional status of secondary school adolescent girls in Nnewi, south east Nigeria. Trop J Obstetrics Gynaecology. 2020;37:482–494.

[24] Adu OB , Falade AM , Nwalutu EJ , et al. Nutritional status of undergraduates in a Nigerian university in south-west Nigeria. Int J Med Med Sciences. 2009;1:318–324.

[25] Afolabi W , Towobola SK , Oguntona CRB , et al. Pattern of fast food consumption and contribution to nutrient intakes of Nigerian university students. Int J Educ Res. 2013;1:1–10.

[26] Agofure O , Odjimogho S , Okandeji-Barry O , et al. Dietary pattern and nutritional status of female adolescents in Amai secondary school, Delta state, Nigeria. Pan Afr Med J. 2021;38:32. 33777300 10.11604/pamj.2021.38.32.15824 PMC7955598

[27] Agoreyo BO , Obuekwe IF. Public health implications of the declining calcium intake in female adolescents from a Nigerian university. J Int Women’s Studies. 2002;4:35–42.

[28] Ajuzie NC , Sanusi RA , Makinde YO. Nutritional status and school performance of primary school children in Ogun state, Nigeria. J Nutr Health Food Science. 2018;6:1–7.

[29] Akinbodewa AA , Adejumo AO , Lamidi OA , et al. Clustering of cardiometabolic risk factors among children and adolescents in a rural community in Ondo, southwest Nigeria. J Trop Pediatrics. 2020;66:366–376. 10.1093/tropej/fmz075 31665517

[30] Akinlade AR , Afolabi WAO , Oguntona EB et al. Prevalence of obesity among adolescents in senior secondary schools in Oyo state, Nigeria. Age. 2014;12:219.

[31] Akinola I , Odugbemi B , Bakare O , et al. Dietary habits, physical activity and sleep pattern among in-school adolescents in Lagos, Nigeria. Ann Health Research. 2022;8:63–73.

[32] Akinyemi O , Ibraheem AG. Assessment of nutritional status of queens college students of Lagos State, Nigeria. Pak J Nutr. 2009;8:937–939.

[33] Ansa VO , Anah MU , Ndifon WO. Soft drink consumption and overweight/obesity among Nigerian adolescents. Global Heart. 2008;3:191–196.

[34] Anyika JU , Uwaegbute AC , Olojede AO , et al. Nutrient intakes of adolescent girls in secondary schools and universities in Abia state of Nigeria. Pak J Nutrition. 2009;8:1596–1602.

[35] Atawodi SE , Aliyu B , Abbas O , et al. Nutritional status of primary school children in Kawo district of Kaduna metropolis, Nigeria. Ann Res Rev Biology. 2015;5:64–70.

[36] Ayogu RN , Nnam NM , Ibemesi O , et al. Prevalence and factors associated with anthropometric failure, vitamin A and iron deficiency among adolescents in a Nigerian urban community. Afr Health Sci. 2016;16:389–398. 27605954 10.4314/ahs.v16i2.7 PMC4994541

[37] Ayogu RNB , Afiaenyi IC , Madukwe EU , et al. Prevalence and predictors of under-nutrition among school children in a rural south-eastern Nigerian community: a cross sectional study. BMC Public Health. 2018;18:1–9. 10.1186/s12889-018-5479-5 PMC5932855 29720136

[38] Ayogu R. Energy and nutrient intakes of rural Nigerian school children: relationship with dietary diversity. Food Nutr Bull. 2019;40:241–253. 31064219 10.1177/0379572119833854

[39] Ayogu RNB , Nwodo CJ. Epidemiological characteristics of hypertension, impaired fasting capillary glucose and their comorbidity: a retrospective cross-sectional population-based study of rural adolescents in southeast Nigeria. BMJ Open. 2021;11:e041481. 10.1136/bmjopen-2020-041481 PMC8103371 33952534

[40] Bamidele B , Oyenike E , Olusegun TA. Dietary pattern and nutritional status of primary school pupils in a south western Nigerian state: a rural urban comparison. Afr J Food Sci 2016;10:203–212.

[41] Charles Shapu R , Ismail S , Ahmad N , et al. Knowledge, attitude, and practice of adolescent girls towards reducing malnutrition in Maiduguri Metropolitan council, Borno state, Nigeria: cross-sectional study. Nutrients. 2020;12:1681. 32512907 10.3390/nu12061681 PMC7353014

[42] Cole AH , Taiwo OO , Nwagbara NI , et al. Energy intakes, anthropometry and body composition of Nigerian adolescent girls: a case study of an institutionalized secondary school in Ibadan. Br J Nutr 1997;77:497–509. 9155501 10.1079/bjn19970052

[43] Darling AM , Sunguya B , Ismail A , et al. Gender differences in nutritional status, diet and physical activity among adolescents in eight countries in sub-Saharan Africa. Trop Med Int Health: TM & IH. 2020;25:33–43. 31693777 10.1111/tmi.13330

[44] Ekekezie OO , Odeyemi KA , Ibeabuchi NM. Nutritional status of urban and rural primary school pupils in Lagos state, Nigeria. West Afr J Medicine. 2012;31:232–237. 23468024

[45] Elizabeth A , Houmsou RS , Soumay R. Assessment of nutritional status of school children in Makurdi, Benue state. Pak J Nutrition. 2009;8:691–694.

[46] Ene-Obong HN , Doh IF , Ikwuagwu OE. Plasma vitamin A and C status of in-school adolescents and associated factors in Enugu state, Nigeria. J Heatlh Popul Nutr. 2003;21:18–25. 12751670

[47] Ene-Obong H , Ibeanu V , Onuoha N , et al. Prevalence of overweight, obesity, and thinness among urban school-aged children and adolescents in southern Nigeria. Food Nutr Bull 2012;33:242–250. 23424890 10.1177/156482651203300404

[48] Eneobong HN. Adolescents living in boarding houses in Nsukka, Enugu state, Nigeria. 2. Quality of school meals and snacks and their contribution to nutrient intake. Ecol Food Nutrition. 1993;30:195–205.

[49] Erinoso HO , Olusanya O , Atinmo T. Nutrient intakes of children in a rural Nigerian community. J Trop Pediatr. 1992;38:329–331. 1844095 10.1093/tropej/38.6.329

[50] Esimai O , Ojofeitimi E. Nutrition and health status of adolescents in a private secondary school in port Harcourt. J Health Sci Journal. 2015;9:4.

[51] Essien E , Emebu PK , Iseh KR , et al. Assessment of nutritional status and knowledge of students from selected secondary schools in Sokoto metropolis, Sokoto state, Nigeria. Afr J Food Agric Nutr Dev 2014;14:2254–2268.

[52] Eze JN , Oguonu T , Ojinnaka NC et al. Physical growth and nutritional status assessment of school children in Enugu, Nigeria. Niger J Clin Practice. 2017;20:64–70. 10.4103/1119-3077.180067 27958249

[53] Fadipe B , Oyelohunnu MA , Olagunju AT , et al. Disordered eating attitudes: demographic and clinico-anthropometric correlates among a sample of Nigerian students. Afr Health Sci. 2017;17:513–523. 29062348 10.4314/ahs.v17i2.27 PMC5637038

[54] Fagbamigbe AF , Adebowale AS , Ajayi I. An assessment of the nutritional status of ART receiving HIV-orphaned and vulnerable children in south-west Nigeria. Heliyon. 2019;5:e02925. 31872116 10.1016/j.heliyon.2019.e02925 PMC6909062

[55] Adekanmbi AF , Obadina OO , Fetuga M , et al. Prevalence of malnutrition and high blood pressure amongst adolescents in semi-urban area of Ogun state south- western Nigeria. Niger Med Practitioner. 2016;69:83–88.

[56] Funke OM , Ajayi A. Determinants of food choices of adolescents in south-western Nigeria. Afr J Food Agric Nutr Dev 2007;7:1–14.

[57] Goon DT , Toriola AL , Shaw BS et al. Anthropometrically determined nutritional status of urban primary schoolchildren in Makurdi, Nigeria. BMC Public Health. 2011;11:1–8. 21974827 10.1186/1471-2458-11-769 PMC3198944

[58] Henry-Unaeze HN , Okonkwo CN. Food consumption pattern and calcium status of adolescents in Nnewi, Nigeria. Pak J Nutrition. 2011;10:317–321.

[59] Ikorok MM , Eka RJ , Ogunjimi LO , et al. Determinants of nutritional behaviour of secondary school students in Akwa Ibom state, Nigeria. Int J Nutr Metabolism. 2012;4:94–99.

[60] Ikujenlola AV , Adekoya TS. Nutritional status and feeding habits of females in public and private universities in Osun state, Southwestern, Nigeria. Heliyon. 2020;6:9. 10.1016/j.heliyon.2020.e05023 PMC7519375 33005810

[61] Iyalomhe SI , Iyalomhe SE , Nwadike IG , et al. Assessment of dietary habits and nutritional status of adolescents in a resource–poor environment in Nigeria. Int J Nutr Food Sciences. 2018;7:121.

[62] Kayode OO , Alabi QK. Food consumption patterns, physical activity and overweight and obesity among undergraduates of a private university in Nigeria. Clin Nutr Experimental. 2020;31:28–34.

[63] Kelvin AA , Sanusi RA. Nutritional status of in-school adolescents in Ekiti state, Nigeria. Glob J Med Public Health. 2016;5:1–11.

[64] Kola-Raji BA , Balogun MR , Odugbemi TO. A comparative study of nutritional status of adolescents from selected private and public boarding secondary schools in Ibadan, south western Nigeria. J Med Tropics. 2017;19:49.

[65] Lateef OJ , Njogu E , Kiplamai F , et al. Determinants of overweight and obesity among adolescent students in public secondary schools in Kwara state, Nigeria. Curr Res Nutr Food Sci Journal. 2016;4:96–106.

[66] Nnanyelugo DO , Okeke EC. Food habits and nutrient intakes of Nigerian university students in traditional halls of residence. J Am Coll Nutr. 1987;6:369–374. 3655159 10.1080/07315724.1987.10720201

[67] Nwokoro SO , Ifada K , Onochie O , Olomu JM. Anthropometric assessment of nutritional status and growth of 10–20 years old individuals in Benin city (Nigeria) metropolis. Pak J Nutrition. 2006;5:117–121.

[68] Ogechi UP , Akhakhia OI , Ugwunna UA. Nutritional status and energy intake of adolescents in Umuahia urban, Nigeria. Pak J Nutrition. 2007;6:641–646.

[69] Ogechi UP. Energy intake, expenditure and body composition of adolescent boys and girls in public boarding secondary schools in Umuahia, Nigeria. Energy. 2012;2:1–7.

[70] Ogunkunle MO , Oludele AS. Food intake and meal pattern of adolescents in school in Ila Orangun, south-west Nigeria. S Afr J Clin Nutrition. 2013;26:188–193.

[71] Ogunsile SE. The effect of dietary pattern and body mass index on the academic performance of in-school adolescents. Int Educ Studies. 2012;5:65–72.

[72] Oguntona CR , Kanye O. Contribution of street foods to nutrient intakes by Nigerian adolescents. Nutr Health. 1995;10:165–171. 7491168 10.1177/026010609501000206

[73] Okeke EC , Nnanyelugo DO. Intrafamilial distribution of food and nutrients in a rural Nigerian population. Ecol Food Nutrition. 1989;23:109–123.

[74] Okoro EO , Ogunbiyi AO , George AO , et al. Association of diet with acne vulgaris among adolescents in Ibadan, southwest Nigeria. Int J Dermatol. 2016;55:982–988. 26749364 10.1111/ijd.13166

[75] Okpokowuruk FS , Akpan MU , Ikpeme EE. Prevalence of hypertension and prehypertension among children and adolescents in a semi-urban area of Uyo metropolis, Nigeria. Pan Afr Med Journal. 2017;28:303. 29854068 10.11604/pamj.2017.28.303.14396 PMC5966115

[76] Olatona FA , Onabanjo OO , Ugbaja RN , et al. Dietary habits and metabolic risk factors for non-communicable diseases in a university undergraduate population. J Health Popul Nutr. 2018;37:21. 30115131 10.1186/s41043-018-0152-2 PMC6097209

[77] Olatona FA , Ogide PI , Abikoye ET , et al. Dietary patterns, nutritional knowledge and status of adolescents in Lagos, Nigeria. Res Square. 2020. DOI: 10.21203/rs.3.rs-18023/v1. PMC10521850 37767409

[78] Olatona FA , Oloruntola OO , Adeniyi OF , et al. Association between breakfast consumption and anthropometrically determined nutritional status of secondary-school adolescents in Lagos, southwest Nigeria. Int J Maternal Child Health AIDS. 2022;11:e503. 10.21106/ijma.503 PMC9083379 35601681

[79] Olorunfemi KA , Aimola IA , Nzelibe HC , et al. The relatiopnship between socio-economic indices and nutritional status of adolescents with sickle cell anaemia at a tertiary hospital in northern Nigeria (2). J Res Basic Clin Sciences. 2019;1.

[80] Olumakaiye MF , Atinmo T , Olubayo-Fatiregun MA. Food consumption patterns of Nigerian adolescents and effect on body weight. J Nutr Educ Behavior. 2010;42:144–151. 10.1016/j.jneb.2008.12.004 20083439

[81] Olumakaiye MF. Adolescent girls with low dietary diversity score are predisposed to iron deficiency in southwestern Nigeria. ICAN: Infant, Child, & Adolescent Nutrition. 2013;5:85–91.

[82] Olumuyiwa SF , Israel O , Oluwemimo O , et al. School feeding programme in Nigeria: the nutritional status of pupils in a public primary school in Ile-Ife, Osun state, Nigeria. Food Nutr Sciences. 2012;3:596–605.

[83] Oroniran OO , Olawuyi YO , Fadupin GT , Takpatore P. Snack and beverage consumption patterns among undergraduates ata private Nigerian university. World Nutr J. 2020;11:42–57.

[84] Omigbodun OO , Adediran KI , Akinyemi JO et al. Gender and rural–urban differences in the nutritional status of in-school adolescents in south-western Nigeria. J Biosocial Sci 2010;42:653–676. 10.1017/S0021932010000234 20529411

[85] Omobuwa O , Alebiosu CO , Olajide FO et al. Assessment of nutritional status of in-school adolescents in Ibadan, Nigeria. S Afr Family Practice. 2014;56:246–250.

[86] Omuemu VO , Oko-Oboh AG. Meal pattern and soft drink consumption among in-school adolescents in Benin-city, Edo state, Nigeria. J Med Biomed Research. 2015;14:72–81.

[87] Omuemu VO , Omuemu CE. The prevalence of overweight and its risk factors among adolescents in an urban city in Edo state. Niger J Clin Practice. 2010;13:128–133. 20499742

[88] Onabanjo OO , Balogun OL. Anthropometric and iron status of adolescents from selected secondary schools in Ogun state, Nigeria. ICAN: Infant, Child, & Adolescent Nutrition. 2014;6:109–118.

[89] Onimawo IA , Ukegbu PO , Asumugha VU , et al. Assessment of anaemia and iron status of school age children (aged 7–12 years) in rural communities of Abia state, Nigeria. AFr J Food Agric Nutr Dev. 2010;10:1–17.

[90] Oninla SO , Owa JA , Onayade AA , et al. Comparative study of nutritional status of urban and rural Nigerian school children. J Trop Pediatrics. 2007;53:39–43. 10.1093/tropej/fml051 17046961

[91] Onofiok N , Nnanyelugo DO , Ukwondi BE. Usage patterns and contribution of fermented foods to the nutrient intakes of low income households in Emene, Nigeria. Plant Foods Hum Nutr. 1996;49:199–211. 8865329 10.1007/BF01093216

[92] Onuoha O , Eme P. Prevalence of overweight and obesity among adolescents in secondary schools in Aba south LGA, Abia state. Ann Nutr Metabolism. 2013;63:1508–1508.

[93] Onyechi UA , Okolo AC. Prevalence of obesity among undergraduate students, living in halls of residence, university of Nigeria, Nsukka Campus, Enugu state. Animal Res Int J. 2008;5:928–931.

[94] Onyiriuka A , Ibeawuchi A , Onyiriuka R. Assessment of eating habits among adolescent Nigerian urban secondary schoolgirls. Sri Lanka J Child Health. 2013;42:20–26.

[95] Onyiriuka AN , Egbagbe EE , Onyiriuka EPA. Snack consumption pattern among adolescent Nigerian urban secondary school girls. Int J Child Adolesc Health. 2013;6:311.

[96] Onyiriuka AN , Umoru DD , Ibeawuchi AN. Weight status and eating habits of adolescent Nigerian urban secondary school girls. S Afr J Child Health. 2013;7:108–111.

[97] Opara DC , Ikpeme EE , Ekanem US. Prevalence of stunting, underweight and obesity in school aged children in Uyo, Nigeria. Pak J Nutrition. 2010;9:459–466

[98] Oranusi S , Galadima M , Umoh VJ , et al. Energy intake and anthropometry: a case study of families in Zaria, Nigeria. Afr J Biotechnology. 2007;6:459–464.

[99] Orisa CAGOW. Diet, physical activity and food consumption pattern of adolescent girls in port Harcourt, Rivers state, Nigeria. Eur J Nutr Food Safety. 2021;13:38–47.

[100] Otekunrin OA , Otekunrin OA. Exploring dietary diversity, nutritional status of adolescents among farm households in Nigeria: do higher commercialization levels translate to better nutrition? Nutr Food Science. 2022;53:500–520.

[101] Otemuyiwa IO , Adewusi SR. Food choice and meal consumption pattern among undergraduate students in two universities in southwestern Nigeria. Nutr Health. 2012;21:233–245. 24620005 10.1177/0260106013510994

[102] Otuneye AT , Ahmed PA , Abdulkarim AA , et al. Relationship between dietary habits and nutritional status among adolescents in Abuja municipal area council of Nigeria. Niger J Paediatrics. 2017;44:128–135.

[103] Samuel FO , Adetunmbi AJ , Ariyo O. Dietary diversity and anthropometric characteristics of in-school adolescents in the university of Ibadan community. WAJFN. 2015;13:56–65.

[104] Samuel FO , Adenekan RA , Adeoye IA , et al. Nutritional status, dietary patterns and associated factors among out-of-school adolescents in Ibadan, Nigeria. World Nutrition. 2021;12:51–64.

[105] Sanusi RA , Wang D , Ariyo O , et al. Food sources of key nutrients, meal and dietary patterns among children aged 4–13 years in Ibadan, Nigeria: findings from the 2019 kids nutrition and health study. Nutrients. 2021;14:200. 35011075 10.3390/nu14010200 PMC8747053

[106] Senbanjo IO , Oshikoya KA , Odusanya OO , et al. Prevalence of and risk factors for stunting among school children and adolescents in Abeokuta, southwest Nigeria. J Health Popul Nutr 2011;29:364. 21957675 10.3329/jhpn.v29i4.8452 PMC3190367

[107] Senbanjo IO , Oshikoya KA , Njokanma OF. Upper arm composition and nutritional status of school children and adolescents in Abeokuta, southwest Nigeria. World J Pediatrics. 2014;10:336–342. 10.1007/s12519-014-0470-4 24599617

[108] Shapu RC , Ismail S , Ahmad N , et al. Food security and hygiene practice among adolescent girls in Maiduguri Metropolitan council, Borno state, Nigeria. Foods. 2020;9:1265. 32927593 10.3390/foods9091265 PMC7555868

[109] Shokunbi OS , Ukangwa NA. Relationship of blood pressure status, dietary factors and serum electrolytes of in-school adolescents in Ilishan-Remo, Ogun state, Nigeria. Afr Health Sciences. 2021;21:1754–1763. 10.4314/ahs.v21i4.32 PMC8889815 35283970

[110] Sholeye OO , Animasahun VJ , Salako AA , et al. Snacking and sweetened beverage consumption among adolescents in Sagamu, southwest Nigeria. Nutr Food Science. 2018;48:442–452

[111] Silva OO , Ayankogbe OO , Odugbemi TO. Knowledge and consumption of fruits and vegetables among secondary school students of Obele community junior high school, Surulere, Lagos state, Nigeria. J Clin Sciences. 2017;14:68.

[112] Tassy M , Eldridge AL , Sanusi RA , et al. Nutrient intake in children 4–13 years old in Ibadan, Nigeria. Nutrients. 2021;13:1741. 34063783 10.3390/nu13061741 PMC8223787

[113] Uba DS , Islam MR , Haque MI , et al. Nutritional status of adolescent girls in a selected secondary school of north-eastern part of Nigeria. Middle na J Rehabil Health. 2020;7:1–7.

[114] Umeokonkwo AA , Ibekwe MU , Umeokonkwo CD , et al. Nutritional status of school age children in Abakaliki metropolis, Ebonyi state, Nigeria. Bmc Pediatrics. 2020;20:114. 32145745 10.1186/s12887-020-1994-5 PMC7060553

[115] Wariri O , Akhimienho KI , Alhassan JAK , et al. Population and individual-level double burden of malnutrition among adolescents in two emerging cities in northern and southern Nigeria: a comparative cross-sectional study. Ann Global Health. 2020;86:1–11. 10.5334/aogh.3093 PMC7747759 33362989

[116] Yunusa I , Ahmad IM , Gidado ZM , et al. Antioxidant vitamins and body mass index in adolescents. Am J Food Sci Nutr Research. 2014;1:60–65.

[117] Orisa CA , Wordu GO. Diet, physical activity and food consumption pattern of adolescent girls in Port Harcourt, Rivers state, Nigeria. Eur J Nutr Food Safety. 2021;13:38–47.

[118] Onyechi UA , Okolo AC. Prevalence of obesity among undergraduate students, living in halls of residence, university of Nigeria, Nsukka campus, Enugu state. Animal Res International. 2008;5:928–931.

[119] Oroniran OO , Olawuyi YO , Fadupin GT , et al. Snack and beverage consumption patterns among undergraduates Ata private Nigerian university. World Nutr J. 2020;11:42–57.

[120] Ansa VO , Anah MU , Ndifon WO. Soft drink consumption and overweight/obesity among Nigerian adolescents. CVD Prev Control. 2008;3:191–196.

[121] Onimawo IA , Ukegbu PO , Asumugha VU , et al. Assessment of anaemia and iron status of school age children (aged 7–12 years) in rural communities of Abia state, Nigeria. AFr J Food Agric Nutr Dev. 2010;10:1–17.

[122] World Health Organization, Food and Agricultural Organization. Diet, Nutrition and the Prevention of Chronic Diseases: Report of a Joint WHO/FAO Expert Consultation. Geneva: WHO; 2003.

[123] Olatona FA , Oloruntola OO , Adeniyi OF , et al. Association between breakfast consumption and anthropometrically determined nutritional status of secondary-school adolescents in Lagos, southwest Nigeria. Int J MCH AIDS. 2022;11:e503. 35601681 10.21106/ijma.503 PMC9083379

[124] Hayashi C , Mehra V. Undernourished and Overlooked: UNICEF Report Sheds Light on Global Nutrition Crisis Faced by Adolescent Girls and Women. UNICEF Data: Monitoring the Situation of Children and Women. New York: UNICEF; 2023.

[125] National Nutrition and Health Survey. Report on the Nutrition and Health Situation of Nigeria. New York: UNICEF; 2018

[126] United Nations Children’s Fund (UNICEF). Undernourished and overlooked: a global nutrition crisis in adolescent girls and women. New York: UNICEF; 2023.

[127] United Nations Office for the Coordination of Humanitarian Affairs. NIGERIA Situation Report. New York: UNICEF; 2023.

[128] United Nations Children’s Fund. Adolescent Data by Country. New York: UNICEF; 2022.

[129] United Nations Children’s Fund, World Health Organization and International Bank for Reconstruction and Development/The World Bank. Levels and trends in child malnutrition: UNICEF/WHO/World Bank Group Joint Child Malnutrition Estimates: Key findings of the 2023 edition. New York: UNICEF and WHO; 2023.

[130] World Health Organization. Global Nutrition Targets 2025: Policy Brief Series (WHO/NMH/NHD/14.2). Geneva: World Health Organization; 2014.

[131] Global Nutrition Report. Country Nutrition Profiles. Bristol: Global Nutrition Report; 2021.

[132] Lain JW , Vishwanath T. A Better Future for All Nigerians: Nigeria Poverty Assessment 2022 (English). Washington, DC: World Bank Group; 2022.

[133] Ene-Obong HN , ChineloAburime L , Alozie YE , et al. Update on the nutrition situation in Nigeria. Nor Afr J Food Nutr Res. 2020;4:S63–S74.

[134] Yunusa I , Ezeanyika LUS. Dietary intake, anthropometry and nutritional status of adolescents in Nigeria: a review. Asian J Sci Research. 2013;6:16.

[135] Oluwasanu AO , Akinyemi JO , Oluwasanu MM , et al. Temporal trends in overweight and obesity and chronic disease risks among adolescents and young adults: a ten-year review at a tertiary institution in Nigeria. PLoS One 2023;18:e0283210. 37018171 10.1371/journal.pone.0283210 PMC10075485

[136] Ochola S , Masibo PK. Dietary intake of schoolchildren and adolescents in developing countries. Ann Nutr Metabolism. 2014;64:24–40. 10.1159/000365125 25341871

[137] Jaacks LM , Vandevijvere S , Pan A , et al. The obesity transition: stages of the global epidemic. Lancet Diabetes Endocrinology. 2019;7:231–240. 30704950 10.1016/S2213-8587(19)30026-9 PMC7360432

