# Supplementary material for: Adolescent nutrition in Nigeria: a systematic review
Source: J Nutr Sci. 2024 Sep 18;13:e38. doi: 10.1017/jns.2024.34 (PMC11428067; doi:10.1017/jns.2024.34)
Supplement: Gabriel et al. supplementary material [file S204867902400034Xsup001.docx]

**Supplementary File**

**Contents**

[Table S1: Search String per Database 2](#_Toc142489243)

[Fig. S1: Map of Nigeria indicating States including the Federal Capital Territory, Abuja with at least one included study 3](#_Toc142489244)

[Table S3: Supporting Supplementary table 2 for the figure 3 4](#_Toc142489245)

[Table S4: Methodological quality assessment of included studies (Cross Sectional Study) 5](#_Toc142489246)

# Table S1: Search String per Database

| **Database** | **Search string used** |
| --- | --- |
| Google scholars | Nutrition* Adolescen* Nigeria* |
| PubMed | ("nigeria"[MeSH Terms] OR "nigeria"[All Fields] OR "nigeria s"[All Fields]) AND ("adolescences"[All Fields] OR "adolescency"[All Fields] OR "adolescent"[MeSH Terms] OR "adolescent"[All Fields] OR "adolescence"[All Fields] OR "adolescents"[All Fields] OR "adolescent s"[All Fields]) AND ("nutrition s"[All Fields] OR "nutritional status"[MeSH Terms] OR ("nutritional"[All Fields] AND "status"[All Fields]) OR "nutritional status"[All Fields] OR "nutrition"[All Fields] OR "nutritional sciences"[MeSH Terms] OR ("nutritional"[All Fields] AND "sciences"[All Fields]) OR "nutritional sciences"[All Fields] OR "nutritional"[All Fields] OR "nutritionals"[All Fields] OR "nutrition"[All Fields] OR "nutritive"[All Fields]) AND ("diet"[MeSH Terms] OR "diet"[All Fields]) |
| Web of science | ((ALL=(Nigeria)) AND ALL=(adolescent)) AND ALL=(nutrition). |

# Fig. S1: Map of Nigeria indicating States including the Federal Capital Territory, Abuja with at least one included study

# Table S3: Supporting Supplementary table 2 for the figure 3

| **Year** | **Author** | **Underweight** | **Wasting** | **Stunting** | **Obesity** | **Thinness** |
| --- | --- | --- | --- | --- | --- | --- |
| 2000 | Abidoye and Akande | 16 | NR | 20 | NR | NR |
| 2007 | Ogechi et al | NR | NR | 65 | NR | NR |
| 2007 | Oninla et al | 61 | 17 | 28 | NR | NR |
| 2008 | Ansa et al | NR | NR | NR | 2 | NR |
| 2009 | Onyechi and Okolo | NR | NR | NR | 21 | NR |
| 2009 | Elizabeth et al | 50 | NR | NR | NR | NR |
| 2010 | Omigbodun et al | 18 | NR | NR | NR | NR |
| 2010 | Onimawo et al | 77 | 56 | 77 | NR | NR |
| 2010 | Opara et al | 32 | NR | 20 | 6 | NR |
| 2010 | Olumakaiye et al | 20 | NR | NR | NR | NR |
| 2011 | Senbanjo et al | NR | NR | 17 | NR | NR |
| 2011 | Goon et al | 43 | NR | 53 | NR | NR |
| 2011 | Henry-Unaeze et al | 44 | NR | NR | NR | NR |
| 2012 | Ekekezie et al | 30 | 18 | 35 | 13 | NR |
| 2012 | Adesina et al | 6 | NR | 5 | 2 | NR |
| 2012 | Ene-Obong et al | NR | NR | NR | 3 | 13 |
| 2012 | Otemuyiwa and Adewusi | 13 | NR | NR | 6 | NR |
| 2013 | Onuoha and Eme | NR | NR | NR | 2 | NR |
| 2014 | Onabanjo and Balogun | 21 | NR | NR | NR | NR |
| 2014 | Abdulkarim et al | NR | 2 | 11 | 3 | NR |
| 2014 | Akinlade et al | NR | NR | NR | 3 | NR |
| 2014 | Senbanjo et al | NR | NR | 75 | NR | 67 |
| 2014 | Essien et al | 28 | NR | NR | NR | NR |
| 2015 | Esimai et al. | 46 | NR | 36 | NR | NR |
| 2015 | Atawodi et al | 35 | NR | 26 | NR | NR |
| 2015 | Samuel et al | NR | NR | 12 | NR | NR |
| 2016 | Bamidele et al | 33 | 15 | NR | NR | NR |
| 2016 | Kelvin et al | 12 | NR | NR | 5 | NR |
| 2016 | Lateef et al | 29 | NR | NR | 0 | NR |
| 2016 | Folashade et al | NR | 27 | 25 | NR | NR |
| 2016 | Ayogu et al | NR | NR | 33 | NR | NR |
| 2017 | Otuneye et al | NR | 2 | 11 | 3 | NR |
| 2017 | Kola-Raji et al | 39 | NR | 3 | 1 | NR |
| 2017 | Eze et al | 1 | 9 | 0 | 4 | NR |
| 2018 | Olatona et al | NR | NR | NR | 5 | NR |
| 2018 | Ayogu et al | 18 | NR | 42 | NR | 20 |
| 2018 | Iyalomhe et al | 24 | NR | NR | 1 | NR |
| 2018 | Ajuzie et al | 46 | NR | NR | NR | NR |
| 2019 | Adeomi et al | 12 | NR | NR | 1 | NR |
| 2019 | Fagbamigbe et al | 18 | NR | 25 | NR | 25 |
| 2019 | Olorunfemi et al | NR | NR | NR | 6 | 42 |
| 2019 | Adebimpe | NR | NR | NR | NR | NR |
| 2020 | Ikujenlola and Adekoya | 15 | NR | NR | 5 | NR |
| 2020 | Umeokonkwo et al | 8 | NR | 20 | 1 | 7 |
| 2020 | Oluyinka et al | 3 | NR | NR | 5 | NR |
| 2020 | Adinma et al | 8 | NR | NR | 1 | NR |
| 2020 | Darling et al | 19 | NR | 16 | NR | NR |
| 2020 | Olatona et al | 5 | NR | NR | 5 | NR |
| 2020 | Uba et al | 36 | NR | NR | NR | NR |
| 2020 | Wariri et al | NR | NR | NR | NR | 7 |
| 2021 | Agofure et al | 47 | NR | NR | NR | NR |
| 2021 | Ayogu et al | NR | NR | NR | NR | NR |
| 2021 | Tassy et al | NR | NR | NR | 2 | 21 |
| 2021 | Samuel et al | NR | NR | 12 | NR | NR |
| 2022 | Olatona et al | NR | NR | NR | 3 | NR |

NR: Not reported.

# Table S4: Methodological quality assessment of included studies

| **Ref.** | **1** | **2** | **3** | **4** | **5** | **6** | **7** | **8** | **Rating** |
| --- | --- | --- | --- | --- | --- | --- | --- | --- | --- |
| (1) Abdulkarim et al., 2014 | Y | N | Y | Y | N | N | Y | N | Some concerns |
| (2) Abidoye and Akande, 2000 | N | N | N | N | N | N | N | N | High Risk |
| (3) Adebimpe, 2019 | N | N | N | N | Y | Y | N | Y | High Risk |
| (4) Adeomi *et al*., 2019 | Y | N | Y | Y | Y | Y | Y | Y | Low Risk |
| (5) Adeomi *et al*., 2022a | Y | N | Y | Y | Y | Y | Y | Y | Low Risk |
| (6) Adeomi *et al*., 2022b | Y | N | Y | Y | Y | Y | Y | Y | Low Risk |
| (7) Adeomi *et al*., 2022c | N | N | Y | Y | Y | Y | Y | Y | Some concerns |
| (8) Adesina et al., 2012 | Y | Y | Y | Y | N | N | Y | N | Some concerns |
| (9) Adinma *et al*., 2020 | N | N | Y | Y | N | N | Y | N | High Risk |
| (10) Adu *et al*., 2009 | Y | N | Y | Y | N | N | Y | N | Some concerns |
| (11) Afolabi et al., 2013 | N | N | Y | N | N | N | Y | N | High Risk |
| (12) Agofure *et al*., 2021 | N | Y | Y | Y | N | N | Y | N | Some concerns |
| (13) Agoreyo *et al*., 2002 | N | N | N | N | N | N | N | N | High Risk |
| (14) Ajuzie *et al*., 2018 | N | N | Y | Y | N | N | Y | N | High Risk |
| (15) Akinbodewa *et al*., 2020 | Y | Y | Y | Y | Y | Y | Y | Y | Low Risk |
| (16) Akinlade *et al*., 2014 | N | Y | Y | Y | N | N | Y | N | Some concerns |
| (17) Akinola *et al*., 2022 | N | Y | Y | Y | Y | Y | Y | Y | Low Risk |
| (18) Akinyemi *et al*., 2009 | N | N | N | N | N | N | N | N | High Risk |
| (19) Ansa *et al*., 2008 | N | N | Y | Y | N | N | Y | N | High Risk |
| (20) Anyika *et al.*, 2009 | N | N | Y | Y | N | N | Y | N | High Risk |
| (21) Atawodi *et al*., 2015 | N | N | Y | Y | N | N | Y | N | High Risk |
| (22) Ayogu *et al*., 2016 | Y | N | Y | Y | Y | Y | Y | Y | Low Risk |
| (23) Ayogu et al., 2018 | Y | N | Y | Y | Y | Y | Y | Y | Low Risk |
| (24) Ayogu *et al*., 2019 | N | N | Y | Y | Y | Y | Y | Y | Some concerns |
| (25) Ayogu *et al*., 2021 | Y | N | Y | Y | Y | Y | Y | Y | Low Risk |
| (26) Bamidele *et al*., 2016 | N | Y | Y | Y | N | N | Y | Y | Some concerns |
| (27) Charles *et al*., 2020 | N | N | Y | Y | Y | Y | Y | Y | Some concerns |
| (28) Cole et al.,1997 | N | N | Y | Y | N | N | Y | N | High Risk |
| (29) Darling *et al*., 2020 | Y | N | Y | Y | Y | Y | Y | Y | Low Risk |
| (30) Ekekezie *et al*., 2012 | N | Y | Y | Y | N | N | Y | N | Some concerns |
| (31) Elizabeth *et al*., 2009 | N | Y | Y | Y | N | N | Y | N | Some concerns |
| (32) Ene-Obong *et al*., 2003 | N | N | Y | Y | Y | Y | Y | Y | Some concerns |
| (33) Ene-Obong *et al*., 2012 | N | N | Y | Y | N | N | Y | N | High Risk |
| (34) Eneobong, 1993 | N | N | Y | Y | N | N | Y | N | High Risk |
| (35) Erinoso et al., 1992 | N | N | Y | Y | N | N | Y | N | High Risk |
| (36) Esimai et al., 2015 | N | Y | Y | Y | Y | Y | Y | Y | Low Risk |
| (37) Essien et al., 2014 | N | N | Y | Y | N | N | Y | N | High Risk |
| (38) Eze *et al*., 2017 | Y | N | Y | Y | N | N | Y | N | Some concerns |
| (39) Fadipe *et al*., 2017 | N | Y | Y | Y | N | N | Y | N | Some concerns |
| (40) Fagbamigbe *et al*., 2019 | N | Y | Y | Y | N | N | Y | Y | Some concerns |
| (41) Folashade *et al*., 2016 | Y | Y | Y | Y | Y | Y | Y | Y | Low Risk |
| (42) Funke and Ajayi , 2007 | N | Y | Y | Y | N | N | Y | N | Some concerns |
| (43) Goon et al., 2011 | Y | N | Y | Y | N | N | Y | N | Some concerns |
| (44) Henry-Unaeze et al., 2011 | N | N | Y | Y | N | N | Y | N | High Risk |
| (45) Ikorok *et al*., 2012 | N | Y | Y | Y | N | N | Y | N | Some concerns |
| (46) Ikujenlola and Adekoya, 2020 | N | N | Y | Y | N | N | Y | N | High Risk |
| (47) Iyalomhe *et al*., 2018 | Y | N | Y | Y | N | N | Y | N | Some concerns |
| (48) Kayode et al., 2020 | Y | N | Y | Y | N | N | Y | N | Some concerns |
| (49) Kelvin and Sanusi, 2016 | N | Y | Y | Y | N | N | Y | N | Some concerns |
| (50) Kola-Raji *et al*., 2017 | N | N | Y | Y | N | N | Y | N | High Risk |
| (51) Lateef *et al*., 2016 | N | N | Y | Y | N | N | Y | N | High Risk |
| (52) Nnanyelugo et al., 1982 | N | Y | Y | Y | N | N | Y | N | Some concerns |
| (53) Nwokoro *et al*., 2006 | N | N | Y | Y | N | N | Y | N | High Risk |
| (54) Ogechi *et al*., 2007 | N | N | Y | Y | N | N | Y | N | High Risk |
| (55) Ogechi *et al*., 2012 | N | N | Y | Y | N | N | Y | N | High Risk |
| (56) Ogunkunle and Oludele, 2013 | N | N | Y | Y | N | N | Y | N | High Risk |
| (57) Ogunsile,2012 | N | Y | Y | Y | Y | Y | Y | Y | Low Risk |
| (58) Oguntona and Kanye, 1995 | N | N | Y | Y | N | N | Y | N | High Risk |
| (59) Okeke et al., 1989 | N | N | Y | Y | N | N | Y | N | High Risk |
| (60) Okoro *et al*., 2016 | N | Y | Y | Y | N | N | Y | N | Some concerns |
| (61) Okpokowuruk *et al.,* 2017 | N | N | Y | Y | Y | Y | Y | Y | Some concerns |
| (62) Olatona *et al*., 2018 | N | N | Y | Y | N | N | Y | N | High Risk |
| (63) Olatona *et al*., 2020 | N | N | Y | Y | N | N | Y | N | High Risk |
| (64) Olatona *et al*., 2022 | N | N | Y | Y | N | N | Y | N | High Risk |
| (65) Olorunfemi *et al*., 2019 | Y | N | Y | Y | N | N | Y | N | Some concerns |
| (66) Olumakaiye *et* al., 2010 | N | N | Y | Y | N | N | Y | N | High Risk |
| (67) Olumakaiye, 2013 | N | N | Y | Y | N | N | Y | N | High Risk |
| (68) Olumuyiwa et al., 2012 | N | N | Y | Y | N | N | Y | N | High Risk |
| (69) Oluyinka *et al*., 2020 | Y | Y | Y | Y | N | N | Y | N | Some concerns |
| (70) Omigbodun *et al*., 2010 | N | N | Y | Y | Y | Y | Y | Y | Some concerns |
| (71) Omobuwa *et al*., 2014 | N | N | Y | Y | Y | Y | Y | N | Some concerns |
| (72) Omuemu and Oko-Oboh, 2015 | N | N | Y | Y | N | N | Y | N | High Risk |
| (73) Omuemu et al., 2010 | N | N | Y | Y | N | N | Y | N | High Risk |
| (74) Onabanjo and Balogun, 2014 | Y | N | Y | Y | Y | Y | Y | Y | Low Risk |
| (75) Onimawo *et al*., 2010 | N | Y | Y | Y | N | N | Y | N | Some concerns |
| (76) Oninla et al., 2007 | Y | N | Y | Y | N | N | Y | N | Some concerns |
| (77) Onofiok et al., 1996 | N | N | Y | Y | N | N | Y | N | High Risk |
| (78) Onuoha and Eme, 2013 | N | N | Y | Y | N | N | Y | N | High Risk |
| (79) Onyechi and Okolo, 2009 | N | N | Y | Y | N | N | Y | N | High Risk |
| (80) Onyiriuka *et al*., 2013 | N | Y | Y | N | N | N | Y | Y | Some concerns |
| (81) Onyiriuka *et al*., 2013 | N | Y | Y | N | Y | Y | Y | Y | Some concerns |
| (82) Onyiriuka *et al*., 2013 | N | Y | Y | N | Y | Y | Y | Y | Some concerns |
| (83) Opara and IEE, 2010 | N | Y | Y | Y | N | N | Y | N | Some concerns |
| (84) Oranusi *et al.,* 2007 | N | N | Y | N | N | N | Y | N | High Risk |
| (85) Orisa and Wordu, 2021 | N | N | Y | N | N | N | Y | N | High Risk |
| (86) Otekunrin and Otekunrin,2022 | N | N | Y | Y | Y | Y | Y | Y | Some concerns |
| (87) Otemuyiwa and Adewusi, 2012 | N | N | Y | N | N | N | Y | N | High Risk |
| (88) Otuneye *et al*., 2017 | N | N | Y | N | Y | Y | Y | Y | Some concerns |
| (89) Samuel *et al.,* 2015 | N | N | Y | N | N | N | Y | N | High Risk |
| (90) Samuel *et al.,* 2021 | N | Y | Y | Y | N | N | Y | N | Some concerns |
| (91) Sanusi *et al*., 2021 | N | N | Y | Y | N | N | Y | N | High Risk |
| (92) Senbanjo *et al*., 2011 | N | N | Y | Y | N | N | Y | Y | Some concerns |
| (93) Senbanjo *et al*., 2014 | N | N | Y | Y | N | N | Y | Y | Some concerns |
| (94) Shapu *et al*., 2020 | N | N | Y | N | Y | Y | Y | Y | Some concerns |
| (95) Shokunbi and Ukangwa, 2021 | Y | Y | Y | N | N | N | Y | N | Some concerns |
| (96) Sholeye *et al*., 2018 | N | Y | Y | N | N | N | Y | N | High Risk |
| (97) Silva et al., 2017 | N | Y | Y | Y | N | N | Y | N | Some concerns |
| (98) Tassy *et al*., 2021 | Y | Y | Y | Y | N | N | Y | N | Some concerns |
| (99) Uba *et al*., 2020 | Y | N | Y | Y | N | N | Y | N | Some concerns |
| (100) Umeokonkwo *et al*., 2020 | N | Y | Y | Y | N | N | Y | Y | Some concerns |
| (101) Wariri *et al.,* 2020 | Y | Y | Y | Y | N | N | Y | N | Some concerns |
| (102) Yunusa et al., 2014 | N | N | Y | Y | N | N | Y | N | High Risk |
